# Long-term effects of plant vs. animal protein supplementation on body composition, muscle strength, physical performance, and cardiometabolic risk factors in adults:a systematic review and meta-analysis of randomized controlled trials

**DOI:** 10.3389/fnut.2026.1813846

**Published:** 2026-04-01

**Authors:** Mohammed Ahmed Yimam, Justin Roberts, Andrea O’Callaghan, Andrew Holwerda, Yvonne Cassidy, Yvette Luiking, Ardy Van Helvoort, Maurizio Muscaritoli

**Affiliations:** 1 Department of Translational and Precision Medicine, Sapienza University of Rome, Rome, Italy; 2 Department of Science, Technology and Society, University School for Advanced Studies IUSS, Pavia, Italy; 3 Department of Public Health, College of Health Science, Woldia University, Woldia, Ethiopia; 4 Danone Research and Innovation Center, Utrecht, Netherlands; 5 Faculty of Science and Engineering, Anglia Ruskin University, Cambridge, United Kingdom; 6 NUTRIM School of Nutrition and Translational Research in Metabolism, Maastricht University Medical Centre, Maastricht, Netherlands

**Keywords:** animal-based protein, body composition, cardio metabolic risk factors, meta-analysis, muscle strength, physical performance, plant-based protein

## Abstract

**Introduction:**

Previous studies have yielded mixed results on the effects of supplementing with plant-based protein (PBP) isolates or concentrates vs. animal-based protein (ABP) on body composition, muscle strength, physical performance, and cardiometabolic risk factors. Consequently, it would be helpful to synthesize pooled evidence from randomized controlled trials (RCTs) on these parameters to assess the efficacy of different protein sources, particularly in the long term.

**Objective:**

To assess the long-term effects (≥ 6 months) of PBP compared to ABP supplementation on body composition, muscle strength, physical performance, and cardiometabolic risk factors in adults aged 18 and older.

**Methods:**

PubMed, Scopus, and Web of Science databases were searched from their inception to 25 February 2025. Relevant studies were also searched by citation tracing. RCTs comparing PBP with ABP supplementation for at least 6 months were included. A random effects model was employed for data pooling. The overall effect estimate was presented using standardized mean difference (SMD) accompanied by a forest plot and prediction intervals.

**Results:**

A total of 18 RCTs involving 1,893 participants met the criteria for inclusion in the systematic review and meta-analysis. In adults aged 18 years and older, long-term supplementation of PBP (largely soy protein) did not show statistically significant differences in lean body mass (LBM), fat mass (FM), total body mass (TBM), upper and lower muscle strength, gait speed (GS), chair stand test (CST), timed up and go (TUG) test, short physical performance battery (SPPB), lipid profiles, blood pressure, fasting blood glucose (FBG), fasting blood insulin (FBI), and homeostatic model assessment (HOMA-IR) for insulin resistance compared with ABP.

**Conclusion:**

Long-term supplementation with PBP, compared with ABP, did not result in differences in body composition, muscle strength, physical performance, or cardiometabolic risk parameters in the adult population. Based on heterogeneity, the data dot provide clear evidence of differences observed between protein sources at present, as long as an adequate quantity of protein is consumed over time.

**Systematic review registration:**

https://www.crd.york.ac.uk/PROSPERO/, Identifier CRD42024604240.

## Introduction

Protein is an indispensable macronutrient for normal cell growth and differentiation, making it fundamental for supporting life (1, 2). It is crucial for anabolism in health and disease (3). In fact, evidence from previous research and meta-analyses demonstrates that protein quality or protein sources (4–9) determined the gains in lean muscle mass and muscle strength following protein ingestion in addition to the protein doses (10) and habitual protein intake (10). Moreover, Cermak et al. (11) and Morton et al. (10) reported that supplementing protein (~1.6 g/kg/day) with prolonged-resistance exercise training (lasting at least 6 weeks) in healthy adults augmented muscle mass and strength (10). Gaining muscle mass and strength are important for physical performance and have implications for older or frail individuals, as they can help lower the risk of falls and fractures while promoting an active, independent lifestyle with associated health and economic advantages (12, 13).

However, previous evidence (5–8, 14) has reported contentious results in the effects of plant-based (PBP) vs. animal-based protein (ABP) intake on body composition, muscle strength, and muscle function (physical performance). These inconsistencies may be related to the inclusion of both short-term (less than 6 months) and long-term studies (5, 6, 14), as well as the diversity of the populations studied (7, 8). Additionally, the presence of resistance exercise in some studies may also influence outcomes (6). Thus, it is important to consider aggregate evidence on skeletal muscle adaptations following long-term protein supplementation (6 months or more), with or without exercise training, as short-term studies may not capture the full hypertrophic response and could underestimate the chronic adaptations that occur with protein intake (15). It is therefore feasible to assess any differences in the efficacy of PBP vs. ABP in the long term.

Although inconsistency existed across studies regarding the link between frailty with cardiometabolic risk factors (dyslipidemia and blood pressure) (16), evidence from the Mendelian randomization study showed a bidirectional causal association between frailty and cardiometabolic diseases, such as coronary artery diseases, stroke, and type 2 diabetes mellitus (T2DM) (17). A meta-analysis of randomized control trials (RCTs) involving individuals with metabolic diseases, including hypertension, overweight, and obesity, indicated that high-quality protein supplementation from sources such as soy, casein, whey, and milk can lead to reductions in total cholesterol (TC), low-density lipoprotein (LDL), and triglycerides (TG) (18). However, the findings were mainly based on low-quality evidence and did not compare PBP vs. ABP sources, thereby requiring consolidated evidence (18). Another meta-analysis of RCTs reported in adult participants with and without hyperlipidemia showed that when plant protein was substituted for animal protein, LDL and non-high-density lipoprotein (HDL) cholesterol was reduced, approximately by 4% (19); however, the evidence was inconsistent and mainly based on short-term trials as well (19).

Additionally, high-quality protein supplementation from soy, casein, whey, and milk reduces systolic blood pressure (SBP) and diastolic blood pressure (DBP) (18); however, interventional studies reported inconsistent results on the effect of PBP vs. ABP on blood pressure (20, 21). Regarding diabetes risk, replacing animal protein with plant protein (per each 20 g) has been associated with a 20% reduction in the risk of T2DM (22). Indeed, this evidence is based on observational studies, so robust evidence from long-term intervention trials is necessary to verify the findings.

Current pooled evidence primarily stems from short-term interventional studies lasting 4–24 weeks. As a result, the long-term effects of PBP compared with ABP supplementation, particularly beyond 6 months, on body composition, muscle strength, physical performance, and cardiometabolic risk factors in adult populations, remain uncertain. This uncertainty is especially pertinent when evaluating typical lifestyle and dietary patterns for sustainable muscle gain, enhanced physical function and performance, and the prevention of cardiometabolic diseases. Addressing this knowledge gap could provide better insights into PBP and ABP supplementation for muscle health and cardiometabolic risks, potentially informing future interventions. Furthermore, PBP may be more sustainable than ABP due to its lower environmental impact (23, 24). Therefore, a systematic review and meta-analysis based on RCTs was conducted to evaluate whether supplementation with PBP is comparable to ABP in the long term on parameters associated with body composition, muscle strength/function, and cardiometabolic risk markers in adults. It was hypothesized that when protein strategies are employed for longer than 6 months, the specific source of protein will be less important, particularly when overall protein quantity is considered.

## Methods

This review was reported in compliance with the Preferred Reporting Items for Systematic Reviews and Meta-Analyses (PRISMA) statement (25) and the Cochrane Handbook for Systematic Reviews of Interventions (26). The protocol of this study was registered with the International Prospective Register of Systematic Reviews (PROSPERO; registration number: CRD42024604240) prior to data extraction.

### Study inclusion criteria

After comprehensive assessment, studies were included if they met the following criteria: (1) studies conducted on adults aged ≥ 18 years; (2) RCTs (parallel or cross-over); (3) intervention group reported PBP supplement (protein concentrate/isolate) with or without an exercise component; (4) control group reported ABP supplement (protein concentrate or isolate) with or without exercise component; (5) the duration of the intervention ≥ 6 months; (6) trials reported at least one outcome parameter for body composition, muscle strength, physical performance, and/or cardiometabolic risk factors indices; (7) trials that provided both the pre- and post-intervention mean and standard deviations to estimate the effect size; (8) studies published in English language; (9) only human studies. Whole food supplements and grey literature were not included in this review. Detailed inclusion criteria are described in Table 1 using the population, intervention, comparison, outcome, time, and study design (PICOTS) framework.

**Table 1:** PICOTS framework for inclusion of the studies.

Parameters	Criteria
Participants (P)	Adults aged ≥ 18 years.
Intervention (I)	PBP supplement with or without exercise.
Comparison (C)	ABP supplement with or without exercise.
Outcomes (O)	Body composition: lean body mass (LBM), fat mass (FM), and total body mass (TBM). Muscle strength: upper extremity muscle strength: hand grip strength (HGS), and bench press; lower extremity muscle strength: knee extension strength (KES), and squat. Physical performance: gait speed (GS), chair stand test (CST), timed up and go (TUG), short physical performance battery (SPPB). Cardiometabolic risk factors: TC, LDL, HDL, TG, fasting blood glucose (FBG), fasting blood insulin (FBI), SBP, DBP, homeostatic model assessment for insulin resistance (HOMA-IR).
Time	At least a 6-month study duration.
Study design (S)	Randomized control trials.

### Search strategy

**Figure 1:**
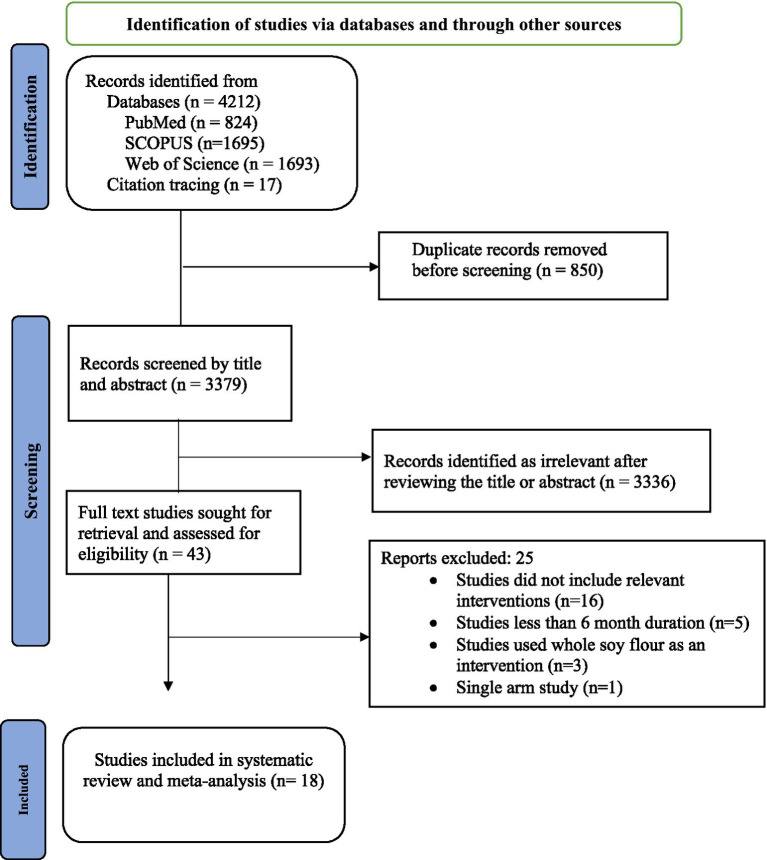
PRISMA flow chart of the literature search process.

PubMed, Scopus, and Web of Science databases were searched from their inception to February 25, 2025. Specific query strings (search terms) were prepared and customized to respective databases. The search terms were developed by combining the following Medical Subject Headings (MeSH) words in PubMed: dietary plant proteins, dietary animal proteins, dietary egg proteins, meat protein, milk protein, body composition, muscle strength, walking speed, gait, physical functional performance, lipoproteins, cholesterol, hyperlipidemia, and triglycerides. We also searched studies through citation tracing. Detailed information regarding the search strategy used to search studies of the present systematic review can be found in Supplementary Table S1. The literature search process is shown in the Preferred Reporting Items for Systematic Reviews and Meta-Analyses (PRISMA) flow chart in Figure 1.

### Data extraction

Before data extraction, studies identified through databases and citation tracing were deduplicated after exporting to EndNote version 20.0.0.14672 software; then the title, abstract, and full text screening of each study was undertaken using the same software. After obtained the eligible articles, the following information were extracted (Table 2): last name of the first author, publication year, country of the study, study design, duration of intervention, health condition of the participants, age, sex, baseline body weight, baseline body mass index (BMI), type and doses of the intervention and control proteins, sample size, and parameters of the studied outcomes (body composition, muscle strength, physical performance, cardiometabolic risk factors). Importantly, the change from baseline values of mean and standard deviation (SD) of each parameter was extracted or calculated, and included in the analysis.

**Table 2:** Characteristics of the included studies.

Author and year of publication	Country	RCT type	Duration (months)	Population	*N*	Type, dosage of intervention, and comparison group	Age in years (rounded)	Sex: M/F	BMI (kg/m^2^)	Baseline daily protein intake, g/day and/ or estimated amount in g/kg/day in brackets	Baseline total energy intake per day (kcal)	Exercise	Main outcomes
Moeller et al., 2003 (36)	USA	Parallel, Double-blind (DB)	6	Healthy perimenopausal women	24	40 g isoflavone rich soy protein isolate ^*^	50 ± 1	F	24.0 ± 3.6	70 ± 18 (1.78)	1,824 ± 411	No	LBM, FM, and TBM
					24	40 g isoflavone poor soy protein isolate^*^	51 ± 20	F	24.2 ± 3.1	73 ± 16 (1.85)	1,828 ± 316		
					21	40 g whey protein	49 ± 11	F	23.9 ± 3.0	70 ± 20 (1.78)	1,769 ± 332		
Li et al., 2021 (40)	China	Parallel, DB	6	Older adults with low lean mass	31	9 g soy protein	69 ± 4	15/16	21.2 ± 2.3	59.6 ± 19.1 (1.10)	1,483 ± 402	No	LBM, TBM, HGS, GS, CST, and SPPB
					31	8 g whey protein	71 ± 4	16/15	21.8 ± 2.0	62.7 ± 20.7 (1.14)	1,558 ± 469		
Kok et al., 2006 (42)	Netherlands	Parallel, DB	12	Healthy postmenopausal women	75	26 g soy protein	67 ± 5	F	26.4 ± 4.1	-	-	No	SPPB
					78	26 g milk protein	67 ± 5	F	26.0 ± 3.4	-	-		
Kjølbæk et al., 2017 (43)	Denmark	Parallel, DB	6	Men and women on weight maintenance	36	45 g soy protein	42 ± 10	8/28	33.3 ± 3.5	(1.00)	1,764 ± 554	No	FM, LBM, TBM, TC, LDL, HDL, TG, FBG, FBI, SBP, DBP, and HOMA-IR
					39	45 g whey protein	42 ± 9	7/32	33.0 ± 3.4	(1.02)	1,741 ± 516		
Jadczak et al., 2021 (44)	Australia	Parallel, DB	6	Prefrail and frail older adults	30	40 g rice protein	73 ± 7	11/19	NR	(1.10)	-	Yes	TBM, LBM, FM, HGS, GS, TUG, and SPPB
					22	40 g whey protein	74 ± 7	7/15	NR	(1.10)	-		
Vupadhyayula et al., 2009 (33)	USA	Parallel, DB	24	Healthy postmenopausal women	48	25 g soy protein without isoflavone*	64 ± 1	F	26.4 ± 0.4	(1.00)	-	No	TUG, HGS, and KES
					57	25 g soy protein with isoflavone*	63 ± 1	F	26.2 ± 0.5	(1.00)	-		
					52	25 g milk protein	64 ± 1	F	26.0 ± 0.4	(1.00)	-		
Volek et al., 2013 (34)	USA	Parallel, DB	9	Healthy men and women	22	20 g soy protein	24 ± 3	11/11	NR	(1.27)	2,032 ± 112	Yes	FM, LBM^†^, TBM, bench press 1 repetition maximum, and squat 1 repetition maximum
					19	21.6 g whey protein	23 ± 4	13/16	NR	(1.27)	2,111 ± 121		
Tomayko et al., 2014 (35)	USA	Parallel, DB	6	Hemodialysis patients	12	27 g soy protein isolate	53 ± 4	F	33.3 ± 2.4	-	-		FM, LBM, TBM, KES, GS, TUG, and CST
					11	27 g whey protein isolate	57 ± 5	F	31.3 ± 2.2	-	-		
Evans et al., 2007 (46)	USA	Parallel, DB	9	Healthy postmenopausal women	10	25.6 g soy protein isolate	64 ± 5	F	NR	-	-	No	FM, LBM^†^, and TBM
					12	25.6 g milk protein isolate	63 ± 5	F	NR	-	-		
					11	25.6 g soy protein isolate	63 ± 5	F	NR	-	-	Yes	FM, LBM^†^, and TBM
					10	25.6 g milk protein isolate	60 ± 5	F	NR	-	-		
Kreijkamp-Kaspers et al., 2004 (41)	Netherlands	Parallel, DB	12	Healthy postmenopausal women	88	25.6 g of soy protein	67 ± 5	F	26.4 ± 4.1	99.64 ± 22.4	2,129 ± 467	No	TC, LDL, HDL, and TG
					87	25.6 g milk protein	67 ± 5	F	25.9 ± 3.5	103.1 ± 22.8	2,153 ± 440		
Campbell et al., 2010 (47)	USA	Parallel, DB	12	Healthy postmenopausal women	35	25 g soy protein	53 ± 6	F	28.6 ± 0.9	75.8 ± 3.6 (1.00)	1,827 ± 82	No	TC, LDL, HDL, and TG
					27	25 g casein	56 ± 5	F	27.3 ± 1.0	64.2 ± 4.1 (0.89)	1,577 ± 95		
Dent et al., 2001 (27)	USA	Parallel, DB	6	Normal and mildly hypercholesterolemic perimenopausal	24	40 g isoflavone-rich soy protein*	51 ± 4	F	24.0 ± 3.6	-	-	No	TC, LDL, HDL, and TG
					24	40 g isoflavone-poor soy protein*	51 ± 5	F	24.2 ± 3.1	-	-		
					21	40 g whey protein	50 ± 3	F	23.9 ± 3	-	-		
Hodis et al., 2011 (45)	USA	Parallel, DB	31	Healthy postmenopausal women	158	25 g soy protein	61 ± 7	F	26.0 ± 6.8	-	-	No	TBM, TC, LDL, HDL^†^, TG, FBG, SBP, and DBP
					153	25 g milk protein	61 ± 7	F	25.8 ± 6.8	-	-		
Liu et al., 2012 (38)	China	Parallel, DB	6	Pre-diabetic postmenopausal women	60	15 g soy protein	56 ± 5	F	24.1 ± 3.8	87.18 ± 29.59 (1.48)	2,203 ± 779	No	TC, LDL, HDL, and TG
					60	15 g milk protein	56 ± 4	F	24.6 ± 3.4	95.55 ± 53.88 (1.59)	2,025 ± 762		
Baum et al., 1998 (48)	USA	Parallel, DB	6	Hypercholesterolemic postmenopausal women	23	40 g soy protein isolate with 56 g isoflavone*	60 ± 9	F	28.2 ± 6.0	-	1,607 ± 431	No	TC, LDL, HDL^†^, and TG
					21	40 g soy protein isolate with 90 g isoflavone*	61 ± 10	F	26.2 ± 4.6	-	-		
					22	40 g casein and non-fat dry milk	61 ± 6	F	29.1 ± 5.2	-	1,670 ± 391		
Liu et al., 2013 (37)	China	Parallel, DB	6	Mild hyperglycemic postmenopausal women	60	15 g soy protein with isoflavone	55 ± 5	F	24.1 ± 3.8	88.1 ± 34.0 (1.48)	2,251 ± 648	No	SBP^†^ and DBP
					60	15 g milk protein	56 ± 4	F	24.6 ± 3.4	81.8 ± 41.1 (1.31)	1,987 ± 641		
Kreijkamp-Kaspers et al., 2005 (20)	Netherlands	Parallel, DB	12	Healthy postmenopausal women	88	25.6 g of soy protein with isoflavone	67 ± 5	F	26.4 ± 4.1	99.64 ± 22.4 (1.40)	1,862 ± 430	No	SBP^†^ and DBP
					87	25.6 g milk protein	67 ± 5	F	26.0 ± 3.4	103.1 ± 22.8 (1.48)	2,141 ± 420		
Liu et al., 2010 (39)	China	Parallel, DB	6	Prediabetic postmenopausal women	60	15 g soy protein with isoflavone	56 ± 5	F	24.1 ± 3.8	81.3 ± 18.4	2,203 ± 779	No	FBG, FBI, and HOMA-IR
					60	15 g milk protein	56 ± 4	F	24.6 ± 3.4	103 ± 22.3	2,025 ± 762		

*The intervention groups were pooled to obtain a single pairwise comparison. NR, not reported; USA, United States of America; LBM, lean body mass; FM, fat mass; TBM, total body mass; HGS, hand grip strength; KES, knee extension strength; GS, gait speed; CST, chair stand test; TUG, timed up and go; SPPB, short physical performance battery; TC, total cholesterol; LDL, low-density lipoprotein; HDL, high-density lipoprotein; TG, triglycerides; FBG, fasting blood glucose; FBI, fasting blood insulin; SBP, systolic blood pressure: DBP, diastolic blood pressure; HOMA-IR, homeostatic model assessment for insulin resistance. ^†^Statistically significant differences between the intervention and control groups.

WebPlot Digitizer, version 5.2, was used to extract the data from the figure, if the corresponding author did not reply to the request for the necessary information (27). We converted TG, TC, HDL, LDL, and FBG from mg/dL to mmol/L to unify the units of measurement (mg/dL divided by 88.57 for TC, 38.67 for HDL and LDL, and 18 for FBG). FBI was converted to pmol/L (FBI conversion: μg/mL divided by 5.807; μIU/mL divided by 6.994).

Screening of titles and abstracts was initially performed by one reviewer (MAY) and verified by another reviewer (JR) to minimize selection bias. Following this, dual independent screening was undertaken by both reviewers (MAY and JR) of full-text articles and data extraction for each parameter. In cases of ambiguity, it was resolved through discussion.

### Evaluation of the risk of bias and certainty of evidence

Two independent reviewers (MAY and JR) evaluated the risk of bias (ROB) of each studied parameters using the Revised Cochrane ROB tool for randomized trials (the Cochrane Risk of Bias 2 [RoB2]) (26), which examines five domains; bias arising from the randomization process, bias due to deviations from intended interventions, bias due to missing outcome data, bias in measurement of the outcome, and bias in selection of the reported result. Results were represented graphically using ROB VISualization (robvis) (28). The certainty of evidence was evaluated using the Grading of Recommendations Assessment, Development, and Evaluation (GRADE) approach by two reviewers (MAY and JR) working independently (29). The risk of bias, inconsistencies, publication bias, indirectness of the evidence, and overall imprecision downgrades the level of evidence (29, 30). The certainty of evidence, rated as high, moderate, low, and very low, was presented through a summary of finding tables (Supplementary Tables S8–S11) which was undertaken using GRADEpro software.^1^ Any discrepancies identified during the risk of bias assessment and certainty of evidence evaluation were solved by discussion.

### Data synthesis and analysis

Mean, SD, and sample size were collated from the included trials. Different conversion formulas, as recommended in the Cochrane Handbook for Systematic Reviews of Interventions (26) were used if the trials did not directly report the change from baseline mean and SD. Standardized mean difference (SMD) with 95% confidence intervals (95% CI) was used to present the pooled effect size for each studied parameter. Moreover, the prediction interval with 95% CI was presented to show the certainty of the pooled effects for future studies. A random effects model was used to estimate the overall effect size, and results were displayed with a forest plot. Between-study heterogeneity was evaluated using the Cochrane Q test and I^2^ statistic, considering an I^2^ value > 50% or a *p*-value of < 0.1 as indicating a heterogeneity existed between studies (26).

To identify possible sources of heterogeneity between protein interventions, subgroup analyses were performed for body composition and lipid profile outcomes (higher number of trials than other studied outcomes) according to the age of the participants (< 60 years vs. ≥ 60 years), protein doses (< 30 g vs. ≥ 40 g), duration of the intervention (< 9 months vs. ≥ 9 months) and exercise (yes vs. no) if applicable. Subgroup analysis by age, protein dose, study quality, and intervention duration was pre-specified; subgroup analysis by exercise was included later. Doi plot (i.e., it is more sensitive and powerful than funnel plot when studies are fewer than 10) with the Luis Furuya-Kanamori (LFK) index (LFK index within ±1 shows no asymmetry) was used to assess small-study effects due to publication bias (31). Rücker’s limit meta-analysis method was used to estimate the adjusted overall effect by correcting the small-study effect due to publication bias. Sensitivity analysis was performed to assess the impact of individual studies on the overall results. All statistical analyses were performed using R software version 4.4.1 (32).

## Results

### Flow of the literature search

As described in the PRISMA flowchart (Figure 1), a total of 4,229 articles were identified by searching from databases and through citation tracing. After the removal of duplicate articles (850), 3,379 articles underwent title and abstract screening, followed by full-text screening of the remaining 43 articles. Finally, 18 eligible trials were systematically reviewed and meta-analyzed (20, 27, 33–48).

### Study characteristics

Eighteen trials fulfilled the study criteria and were included in the systematic review and meta-analysis, and their detailed descriptions are provided in Table 2. To narrate, the included trials were published from 1998 to 2021 and involved a total of 1893 participants, with sample sizes ranging from 10 to 158 individuals. The ages of participants spanned from 23 to 74 years, 9 trials enrolled participants aged < 60 years (27, 34–39, 43, 47), and 9 trials included participants aged ≥ 60 years (20, 33, 40–42, 44–46, 48).

The baseline body mass index (BMI) of the participants ranged from 21.0 to 33.3 kg/m^2^. All studies were parallel, double-blind RCTs. Geographically, 9 trials were conducted in the USA (27, 33–36, 45, 47, 48); 4 trials in China (37–40); 3 trials in the Netherlands (20, 41, 42); 1 trial in Australia (44), and 1 trial in Denmark (43). The length of interventions ranged from 6 to 31 months; 10 studies lasted < 9 months (27, 35–40, 43, 44), whereas 8 studies lasted ≥ 9 months (20, 33, 34, 41, 42, 45–47).

Fourteen trials were conducted with female participants (20, 27, 33, 35–39, 41, 42, 45–48), either peri or postmenopausal women (20, 27, 33, 35–39, 41, 42, 45–48), and 4 trials enrolled both male and female participants (34, 40, 43, 44). Four trials were conducted among older adults with low lean mass (40), hemodialysis patients (35), individuals on weight maintenance (43), and frail older adults (44).

Regarding the intervention groups, 17 trials supplemented participants with soy protein (20, 27, 33–43, 45–48), and 1 trial used rice protein (44). Concerning the control groups, 9 trials supplemented participants with milk protein (20, 33, 37–39, 41, 42, 45, 46), 7 with whey protein (27, 34–36, 40, 43, 44), 1 with casein (47), and 1 with casein and milk (48). Three trials included exercise along with the protein supplements (34, 44, 46). The daily protein doses in the trials ranged from 8 to 45 g; 13 trials used < 30 g, and the remaining trials used ≥ 40 g. The parameters LBM, FM, TBM, upper extremity muscle strength, lower extremity muscle strength, GS, TUG, CST, SPPB, SBP, DBP, FBG, FBI, and HOMA-IR were evaluated in seven (34–37, 40, 43, 44, 46), six (34–36, 43, 44, 46), eight (34–36, 40, 43–46), four (33, 34, 40, 44), three (33–35), three (35, 40, 44), three (33, 35, 44), two (35, 40, 44), three (40, 42, 44), four (37, 42, 43, 45), four (37, 42, 43, 45), three (39, 43, 45), two (39, 43), and two trials (39, 43), respectively. Moreover, each lipid profile parameter (TC, LDL, HDL, and TG) was evaluated in seven trials (27, 38, 41, 43, 45, 47, 48).

### Long-term effect of PBP vs. ABP supplementation on parameters of body composition (LBM, FM, and TBM)

#### LBM

**Figure 2:**
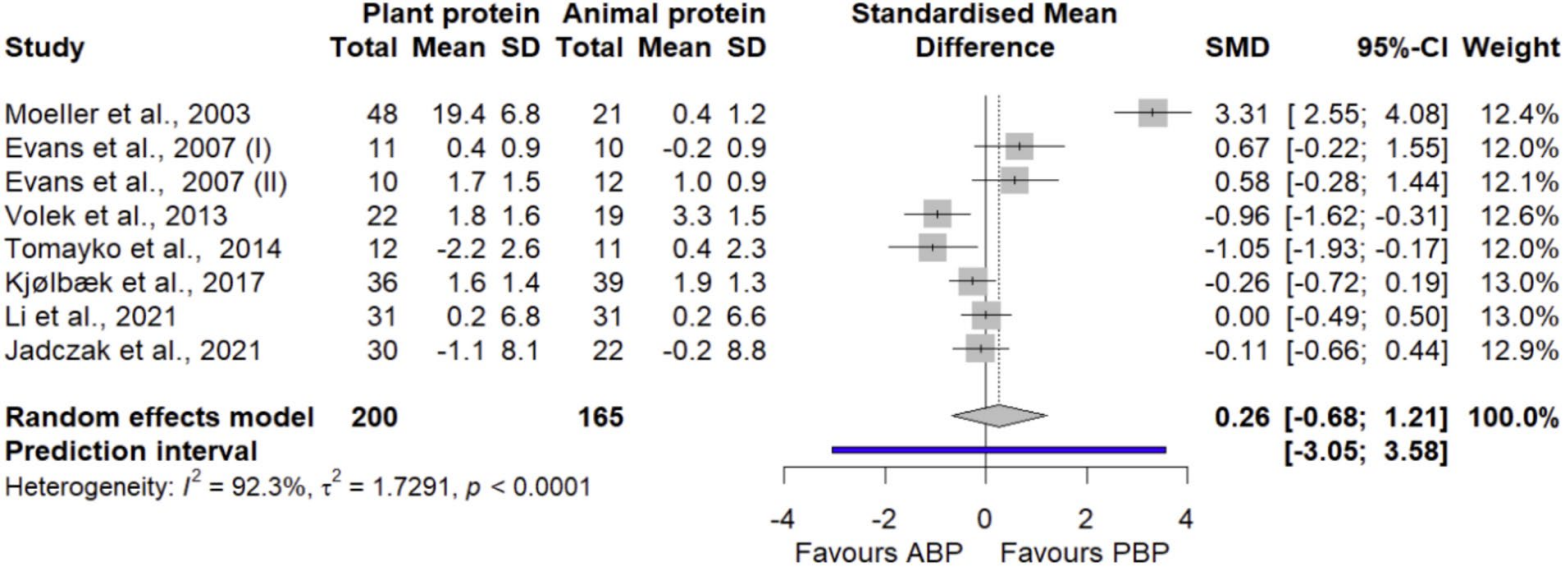
Forest plot of the long-term effect of PBP vs. ABP supplementation on LBM in adults, using the random effects model. “Evans et al., 2007 (I)” indicates groups received both protein supplement and exercise. “Evans et al., 2007 (II)” indicates groups received only protein supplement.

A total of 8 effect sizes from 7 trials (34–36, 40, 43, 44, 46) with 365 participants were pooled to estimate LBM. Figure 2 shows that PBP supplementation did not exhibit any significant difference in LBM when compared to ABP (SMD = 0.26 [95% CI: −0.58 to 0.9; *p*-value = 0.58]). Between-study heterogeneity was significant (I^2^ = 92.3%, *p* < 0.0001). The findings from subgroup analyses showed that PBP intake did not result in any significant differences in LBM by age, protein dose, study quality, duration of intervention, and exercise (Supplementary Table S2). In the sensitivity analysis, the non-significance of the overall effect size was maintained even though the overall effect size (SMD = −0.18 [95% CI: −0.6 to 0.24; I^2^ = 66.4%]) depended on a study by Moeller et al. (36) (Supplementary Table S4). The LFK index value of 1.01 showed a small-study effect, possibly consistent with publication bias. Rücker’s limit meta-analysis method showed the adjusted overall estimate was non-significant (SMD = −0.32 [95% CI: −2.64 to 1.99; *p*-value = 0.78]).

#### FM

**Figure 3:**
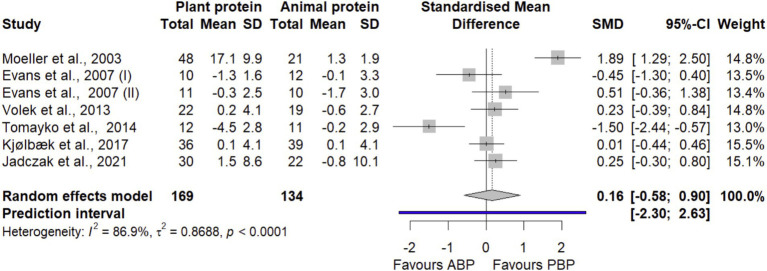
Forest plot of the long-term effect of PBP vs. ABP supplementation on FM in adults, using the random effects model. “Evans et al., 2007 (I)” indicates groups received both protein intervention and exercise. “Evans et al., 2007 (II)” indicates groups received only protein intervention.

Pooling 7 effect sizes from 6 trials (34–36, 43, 44, 46) with 303 participants, the study showed that PBP supplementation did not show significant differences in FM compared with ABP supplementation (SMD = 0.16 [95% CI: −0.58 to 0.90; *p*-value = 0.66], Figure 3). Heterogeneity of studies was significant (I^2^ = 86.9%, *p*-value < 0.0001). Subgroup analyses showed that PBP supplementation did not show any significant differences in FM by age, protein dose, study quality, length of intervention, and exercise compared with ABP (Supplementary Table S2). According to the sensitivity analysis, removing a study by Moeller et al. (36) decreased between-study heterogeneity by 20% without changing the non-significance of the overall effect size (SMD = −0.10 [95% CI: −0.57 to 0.38; I^2^ = 67.02%], Supplementary Table S4). No evidence of a small-study effect, possibly consistent with publication bias, was observed.

#### TBM

**Figure 4:**
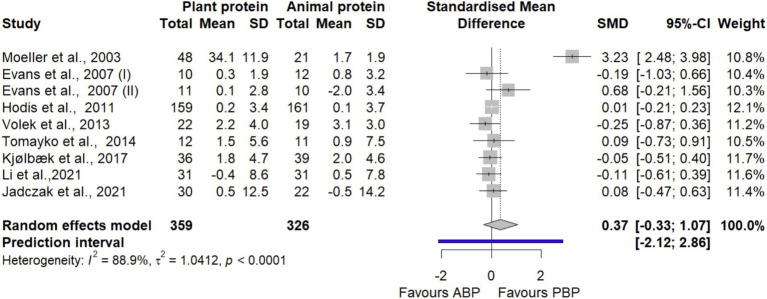
Forest plot of the long-term effect of PBP vs. ABP supplementation on TBM in adults, using the random effects model. “Evans et al., 2007 (I)” indicates groups received both protein supplement and exercise. “Evans et al., 2007 (II)” indicates groups received only protein supplement.

In a meta-analysis combining data from 9 effect sizes from 8 trials (34–36, 40, 43–46) with 685 participants, it was found that PBP supplementation did not show any significant differences (SMD = 0.37 [95% CI: −0.33 to 1.07; *p*-value = 0.30]) on TBM compared with ABP (Figure 4). Significant heterogeneity was detected (I^2^ = 85.9%, *p*-value < 0.0001). Based on subgroup analyses (Supplementary Table S2), no significant subgroup differences existed between protein sources stratified by age, protein dose, study quality, and length of intervention, and exercise. Findings from sensitivity analysis showed that the pooled estimate on TBM remained non-significant even after each trial was removed one by one from the analysis (Supplementary Table S4).

The LFK index value of 1.36 showed evidence of a small-study effect, possibly consistent with publication bias. Rücker’s limit meta-analysis method showed that the adjusted overall estimate was non-significant (SMD = −0.18 [95% CI: −1.61 to 1.24; *p*-value = 0.80]).

### Long-term effects of PBP vs. ABP supplementation on muscle strength, and physical performance

#### Upper extremity muscle strength

**Figure 5:**
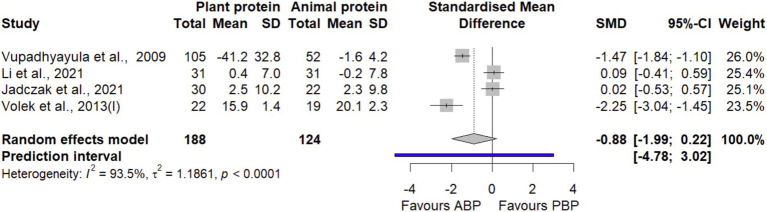
Forest plot of the long-term effect of PBP vs. ABP supplementation on upper extremity muscle strength in adults, using the random effects model. “Volek et al., 2013 (I)” denotes the study reported bench press data.

Combined effect estimates obtained by pooling 4 effect sizes with 312 participants (Figure 5) suggested that PBP supplementation did not show significant differences on upper extremity muscle strength compared with ABP (SMD = −0.88 [95% CI: −1.99 to 0.22; *p*-value = 0.11]). Between-study heterogeneity was significant (I^2^ = 93.5%, *p* < 0.0001). Based on the findings of the sensitivity analysis, none of the studies showed significance on the pooled estimate (Supplementary Table S5). The LFK index (−3.14) showed evidence of a small-study effect, possibly consistent with publication bias. When corrected for small-study effects based on Rücker’s limit meta-analysis method, the overall estimate remained non-significant (SMD = 0.05 [95% CI: −2.25 to 2.35; *p*-value = 0.96]).

#### Lower extremity muscle strength

**Figure 6:**
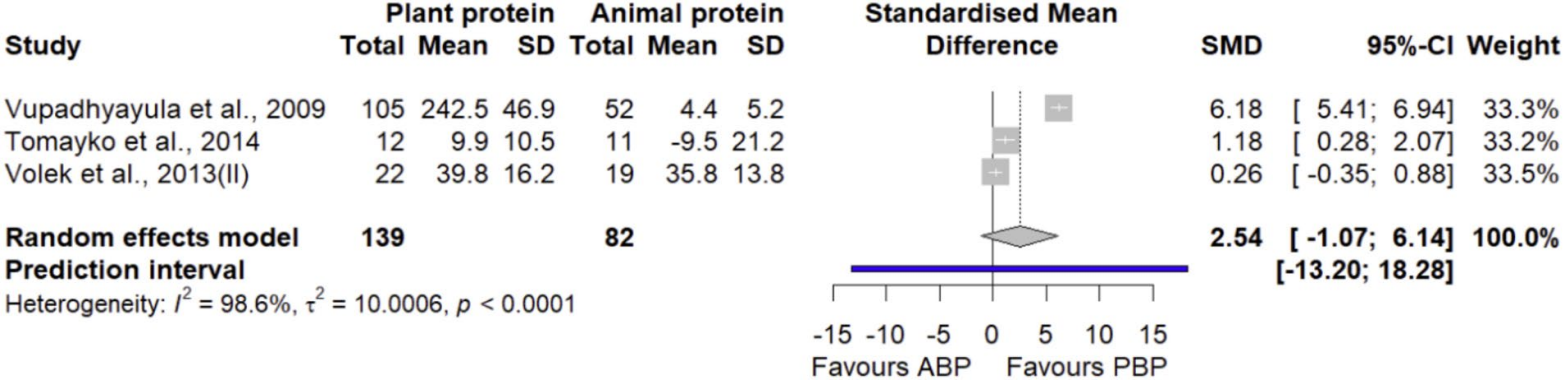
Forest plot of the long-term effect of PBP vs. ABP supplementation on lower extremity muscle strength in adults, using the random effects model. “Volek et al., 2013 (II)” denotes the study reported squat exercise data.

Pooled effect estimates obtained by combining 3 effect sizes with 221participants showed that PBP supplementation did not show significant differences in lower extremity muscle strength compared with ABP (SMD = 2.54 [95% CI: −1.07 to 6.14; *p*-value = 0.16], Figure 6). Between-study heterogeneity was significant (I^2^ = 98.6%, *p* < 0.0001). According to the sensitivity analysis, removing a study by Vupadhyayula et al. (33) decreased between-study heterogeneity by 38% without affecting the non-significance of the overall effect size (SMD = 0.64 [95% CI: −0.21 to 1.49; I^2^ = 60.8%], Supplementary Table S5). The LFK index of 1.42 showed evidence of a small-study effect, possibly consistent with publication bias. When corrected for small-study effects based on Rücker’s limit meta-analysis method, the overall estimate remained non-significant (SMD = 1.33 [95% CI: −11.6 to 14.3; *p*-value = 0.83]).

#### GS

**Figure 7:**
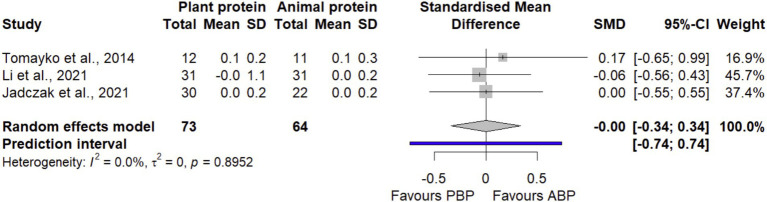
Forest plot of the long-term effect of PBP vs. ABP supplementation on GS in adults, using the random effects model.

Pooling data from 3 effect sizes (35, 40, 44) with 137 participants found that PBP supplementation did not show any significant differences (SMD = −0.00 [95% CI: −0.34 to 0.34; *p*-value = 0.99]) on GS compared with ABP (Figure 7). Between-study heterogeneity was negligible (I^2^ = 0.0%, *p* = 0.89). The LFK index of 1.62 showed evidence of a small-study effect, possibly consistent with publication bias. When corrected for small-study effects based on Rücker’s limit meta-analysis method, the overall estimate remained non-significant (SMD = − 0.4 [95% CI: −2.12 to 1.31; *p*-value = 0.64]).

#### TUG

**Figure 8:**
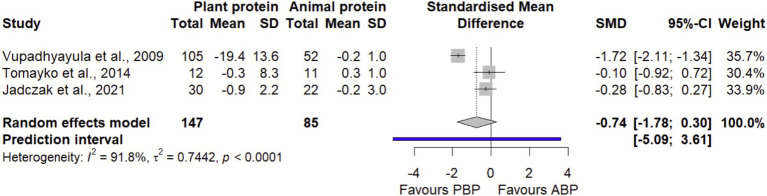
Forest plot of the long-term effect of PBP vs. ABP supplementation on TUG in adults, using the random effects model.

In a meta-analysis combining data from 3 effect sizes with 232 participants, it was found that PBP supplementation did not show any significant differences (SMD = −0.74 [95% CI: −1.78 to 0.30; *p*-value = 0.16]) on TUG compared with ABP (Figure 8). Significant between-study heterogeneity was detected (I^2^ = 91.8%, *p*-value < 0.0001). According to the sensitivity analysis, the pooled effect remained non-significant with negligible heterogeneity (I^2^ = 0.0%, *p*-value < 0.72) after removing a study by Vupadhyayula et al., 2009 (33) (Supplementary Table S5). The LFK index of 4.55 showed evidence of a small-study effect, possibly consistent with publication bias. When corrected for small-study effects based on Rücker’s limit meta-analysis method, the overall estimate showed non-significant (SMD = − 1.7 [95% CI: −3.8 to 0.30; *p*-value = 0.09]).

#### CST

**Figure 9:**
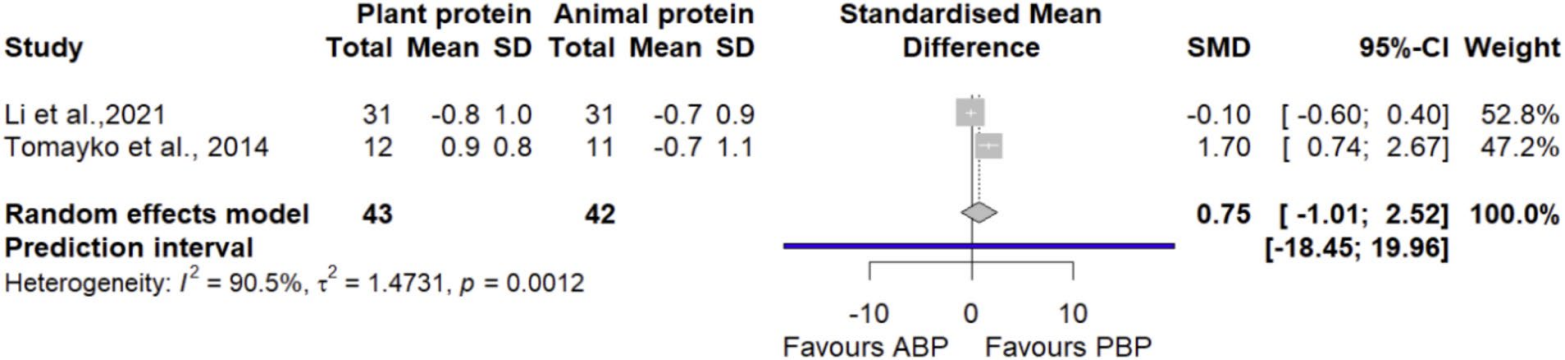
Forest plot of the long-term effect of PBP vs. ABP supplementation on CST in adults, using the random effects model.

In a meta-analysis combining data from 2 effect sizes with 85 participants, it was found that PBP supplementation did not show any significant differences (SMD = 0.75 [95% CI: −1.01 to 2.52; *p*-value = 0.40]) on CST compared with ABP (Figure 9). Significant between-study heterogeneity was detected (I^2^ = 90.5%, *p*-value < 0.0001). The overall effect size depended on study of Li et al. (40), such that, by excluding this study in sensitivity analysis, there were a large and significant effect (SMD = 1.64 [95% CI: 0.70 to 2.59; I^2^ = 0.0%]) of PBP supplementation on chair stand test compared with ABP (Supplementary Table S5). The LFK index of 3.13 showed evidence of a small-study effect, possibly consistent with publication bias. Rücker’s limit meta-analysis method showed the adjusted overall estimate was non-significant (SMD = −0.77 [95% CI: −4.24 to 2.69; *p*-value = 0.66]).

#### SPPB

**Figure 10:**
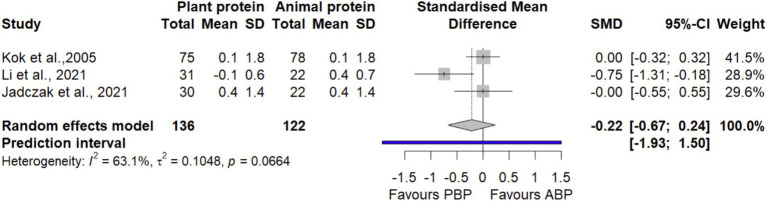
Forest plot of the long-term effect of PBP vs. ABP supplementation on SPPB in adults, using the random effects model.

The pooled effect size of 3 trials (40, 42, 44) involving 258 participants suggested that PBP supplementation had no statistically significant effect on SPPB compared with ABP (SMD = −0.22 [95% CI: −0.67 to 0.24; *p*-value = 0.35], Figure 10). Heterogeneity was observed between studies (I^2^ = 63.1%, *p* = 0.06). After removing the study by Li et al., 2021 (40) in the sensitivity analysis (Supplementary Table S5), the overall effect remains non-significant without detecting heterogeneity (I^2^ = 0.0%, *p* = 1.00). The LFK index of −2.65 showed evidence of a small-study effect, possibly consistent with publication bias. Rücker’s limit meta-analysis method showed the adjusted overall estimate was non-significant (SMD = 0.24 [95% CI: −0.9 to 1.3; *p*-value = 0.68]).

### Long-term effects of PBP vs. ABP supplementation on lipid profiles (TC, LDL, HDL, and TG)

#### TC

**Figure 11:**
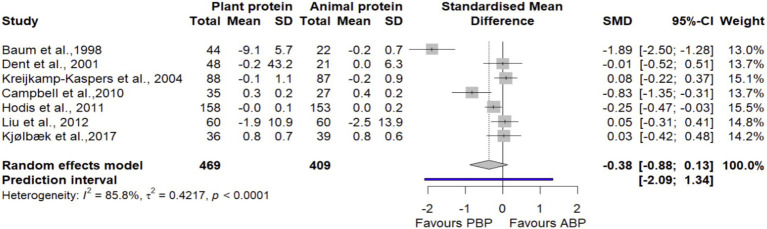
Forest plot of the long-term effect of PBP vs. ABP supplementation on total cholesterol (TC) in adults, using the random effects model.

In a meta-analysis combining data from 7 trials (27, 38, 41, 43, 45, 47, 48) with 878 participants, it was found that PBP supplementation had no statistically significant difference on TC compared with ABP (SMD = −0.38 [95% CI: −0.55 to 0.13; *p*-value = 0.14], Figure 11). Heterogeneity was observed between studies (I^2^ = 85.8%, *p* < 0.0001). The findings from subgroup analyses showed that PBP supplementation compared with animal protein did not result in any significant subgroup differences on TC by age, protein dose, study quality, and duration of intervention (Supplementary Table S3). Based on the sensitivity analysis, removing a study by Baum et al., 1998 (48) decreased between-study heterogeneity by 30% without changing the non-significance of the overall effect size (SMD = −0.13 [95% CI: −0.35 to 0.10; I^2^ = 55.81%], Supplementary Table S6). The LFK index of −2.24 showed evidence of a small-study effect, possibly consistent with publication bias. Rücker’s limit meta-analysis method showed the adjusted overall estimate was non-significant (SMD = −0.48 [95% CI: −0.58 to 1.55; *p*-value = 0.37]).

#### LDL

**Figure 12:**
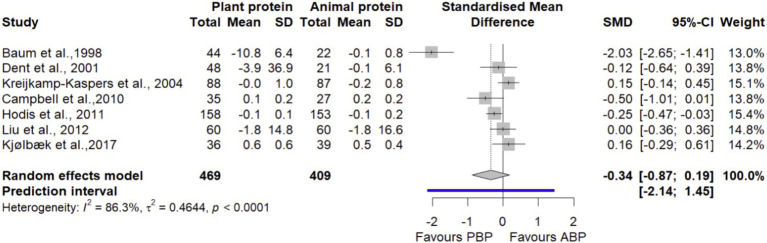
Forest plot of the long-term effect of PBP vs. ABP supplementation on LDL in adults, using the random effects model.

Pooling 7 trials (27, 38, 41, 43, 45, 47, 48) with 878 participants showed that PBP supplementation did not show significant differences in LDL compared with ABP (SMD = −0.34 [95% CI: −0.87 to 0.19; *p*-value = 0.21], Figure 12). Heterogeneity of studies was significant (I^2^ = 86.3%, *p*-value < 0.0001). Subgroup analyses showed that PBP supplementation did not show any significant subgroup differences on LDL by age, protein dose, study quality, and length of intervention compared with ABP (Supplementary Table S3). According to the sensitivity analysis, removing a study by Baum et al., 1998 (48) decreased between-study heterogeneity without changing the non-significance of the overall effect size (SMD = −0.08 [95% CI: −0.27 to 0.14; I^2^ = 39.49%], Supplementary Table S6). The LFK index of −1.37 showed evidence of a small-study effect, possibly consistent with publication bias. Rücker’s limit meta-analysis method showed the adjusted overall estimate was non-significant (SMD = 0.55 [95% CI: −0.54 to 1.65; *p*-value = 0.32]).

#### HDL

**Figure 13:**
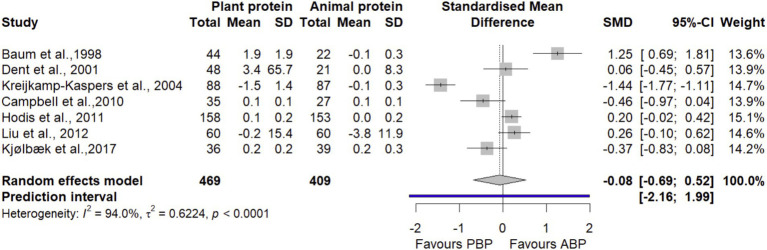
Forest plot of the long-term effect of PBP vs. ABP supplementation on HDL in adults, using the random effects model.

In a meta-analysis combining data from 7 trials (27, 38, 41, 43, 45, 47, 48) with 878 participants, it was found that PBP supplementation had no statistically significant differences on HDL compared with ABP (SMD = −0.08 [95% CI: −0.69 to 0.52; *p*-value = 0.78], Figure 13). Heterogeneity was observed between studies (I^2^ = 94%, *p* < 0.001). The findings from subgroup analyses did not show any significant differences in HDL by age, protein dose, study quality and duration of intervention between protein sources (Supplementary Table S3). Based on the sensitivity analysis, removing a study by Kreijkamp-Kaspers et al. (41) decreased between-study heterogeneity by 7% without changing the non-significance of the overall effect size (SMD = −0.50 [95% CI: −0.31 to 0.60; I^2^ = 86.9%], Supplementary Table S6). The LFK index of −0.76 showed no evidence of a small-study effect, possibly consistent with publication bias.

#### TG

**Figure 14:**
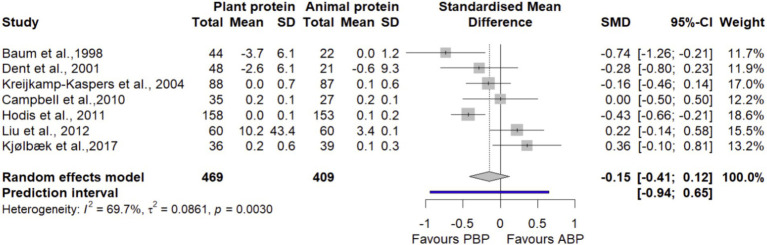
Forest plot of the long-term effect of PBP vs. ABP supplementation on TG in adults, using the random effects model.

Pooling 7 trials (27, 38, 41, 43, 45, 47, 48) with 878 participants showed that PBP supplementation did not show significant differences in TG compared with ABP (SMD = −0.15 [95% CI: −0.41 to 0.12; *p*-value = 0.28], Figure 14). Heterogeneity of studies was significant (I^2^ = 69.7%, *p*-value < 0.001). According to the sensitivity analysis, removing a study by Kjolbaek et al. (43) decreased between-study heterogeneity along with changing the overall effect estimate (SMD = −0.22 [95% CI: −0.47 to −0.03; I^2^ = 63.4%], Supplementary Table S6). Subgroup analyses also showed that PBP supplementation had a smaller reduction effect on TG in trials that had participants aged ≥ 60 years. No significant subgroup differences on TG observed by protein doses, study quality, and length of interventions between the protein sources (Supplementary Table S3). The LFK index of 1.45 showed evidence of a small-study effect, possibly consistent with publication bias. Rücker’s limit meta-analysis method showed the adjusted overall estimate was non-significant (SMD = −0.14 [95% CI: −0.75 to 0.46; *p*-value = 0.64]).

### Long-term effects of PBP vs. ABP supplementation on blood pressure, FBG, FBI and HOMA-IR

#### SBP

**Figure 15:**
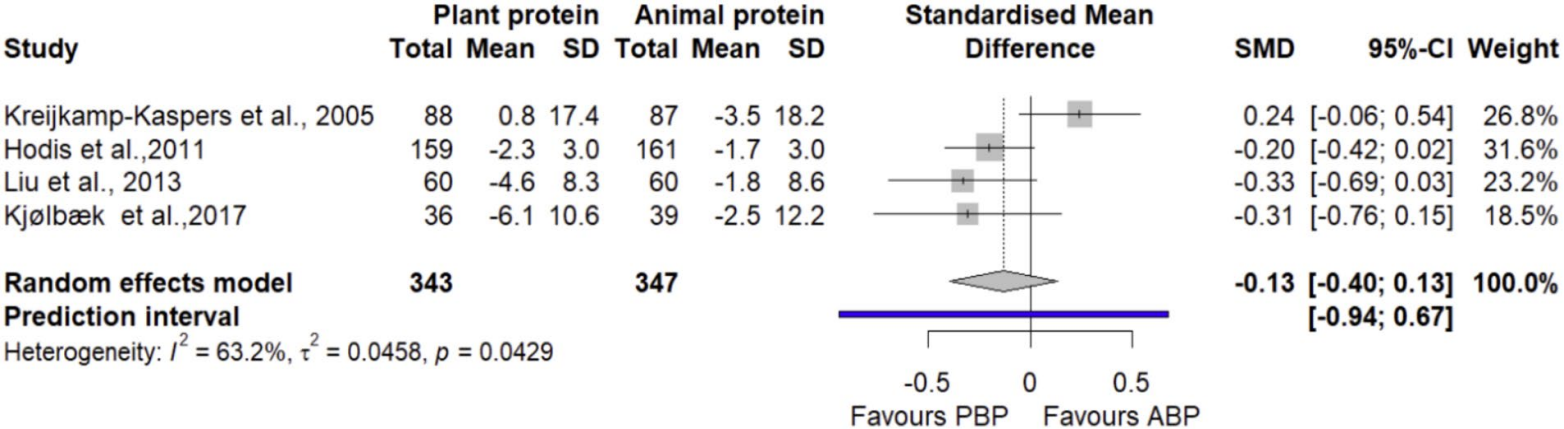
Forest plot of the long-term effect of PBP vs. ABP supplementation on SBP in adults, using the random effects model.

In a meta-analysis combining data from 4 trials (20, 37, 43, 45), it was suggested that PBP supplementation did not show any significant differences (SMD = −0.13 [95% CI: −0.4 to 0.13; *p*-value = 0.33]) on SBP compared with ABP (Figure 15). Moderate heterogeneity was detected (I^2^ = 63.2%, *p*-value = 0.04). The overall effect size depended on Kreijkamp-Kaspers et al., 2005 (20) such that, by excluding this study in sensitivity analysis, there was a smaller reduction effect (SMD = −0.24 [95% CI: −0.42 to −0.07]) of SBP by PBP (Supplementary Table S7). The LFK index of −0.04 showed no evidence of a small-study effect, possibly consistent with publication bias.

#### DBP

The finding from pooling 4 trials (20, 37, 43, 45) found that PBP supplementation did not show any significant differences (SMD = −0.10 [95% CI: −0.30 to 0.10; *p*-value = 0.33]) on DBP compared with ABP (Supplementary Figure 1A). Between-study heterogeneity might not be important (I^2^ = 31.8%, *p*-value = 0.32). After removing Kreijkamp-Kaspers et al., 2005 (20) in the sensitivity analysis, PBP supplementation resulted in a smaller reduction in DBP (SMD = −0.20 [95% CI: −0.37 to −0.03], Supplementary Table S7). The LFK index of 1.27 showed evidence of a small-study effect, possibly consistent with publication bias. Rücker’s limit meta-analysis method showed the adjusted overall estimate remained non-significant (SMD = −0.14 [95% CI: −0.64 to 0.36; *p*-value = 0.58]).

#### FBG

Pooled effect estimates obtained by pooling 3 trials (39, 43, 45) with 668 participants (Supplementary Figure 1B) confirmed that PBP supplementation did not show significant differences on FBG compared with ABP (SMD = 0.24 [95% CI: −0.32 to 0.79; *p*-value = 0.40]). Between-study heterogeneity was significant (I^2^ = 88.8%, *p* < 0.0001). Based on the findings from the sensitivity analysis (Supplementary Table S7), removing a study by Hodis et al. (45) resulted in 0.0% between-study heterogeneity without changing the non-significance of the overall effect result (SMD = −0.04 [95% CI: −0.32 to 0.24; I^2^ = 0.0%]). The LFK index of −4.13 showed a small-study effect/asymmetry, possibly consistent with publication bias. Rücker’s limit meta-analysis showed the adjusted overall estimates for FBG remained non-significant (SMD = 1.03 [95% CI: −0.27 to 2.34; *p*-value = 0.12]).

#### FBI and HOMA-IR

Overall estimate obtained by combining 2 trials (39, 43) showed that PBP did not show significant differences in FBI (SMD = 0.12 [95% CI: −0.16 to 0.40; *p*-value = 0.40]) and HOMA-IR (SMD = 0.18 [95% CI: −0.1 to 0.46; *p*-value = 0.21]) compared with ABP (Supplementary Figures 1C,D). Between-study heterogeneity for both parameters (FBI and HOMA-IR) were 0.0%. Moreover, the LFK index of −2.23 was observed for FBI and HOMA-IR, suggesting a small-study effects/asymmetry possibly consistent with publication bias. However, the Rücker’s limit meta-analysis showed the adjusted overall estimates for FBI (SMD = 0.69 [95% CI: −1.56 to 2.95; *p*-value = 0.54]), and HOMA-IR (SMD = 0.93 [95% CI: −1.34 to 3.22; *p*-value = 0.42]) had remained non-significant.

### Risk of bias results and grading of evidence

The findings of the risk of bias assessment using the ROB 2 tool revealed that 8 trials had some concerns of bias, 2 trials had a high risk of bias, and 8 trials had a low risk of bias (Supplementary Figure 1). According to the GRADE methodology, the studied outcomes were graded as high, moderate, low, and very low certainty of evidence. Supplementary Tables S8–S11 provide information on the GRADE rating method. For TC, LDL, TG, and FBG, the certainty of the evidence was rated low due to downgrades for serious inconsistency and suspected publication bias. SBP and HDL were rated as moderate due to downgrades for serious inconsistency. DBP is rated as moderate due to downgrades for suspected publication bias. The certainty of the evidence was rated as low for LBM, FM, and TBM due to downgrades for serious inconsistency and imprecision. CS, FBI, and HOMA-IR were rated low due to downgrades for serious imprecision and suspected publication bias. Upper extremity muscle strength, lower extremity muscle strength, GS, TUG, and SPPB were rated very low due to downgrades for serious inconsistency, serious imprecision, and suspected publication bias.

## Discussion

To our knowledge, this is the first systematic review and meta-analysis which has considered assessment of the long-term effect (6 months and above) of PBP vs. ABP supplementation in the parameters of body composition (LBM, FM, TBM), muscle strength and physical performance, and cardiometabolic risk factors (TC, LDL, HDL, TG, SBP, DBP, FBG, FBI, and HOMA-IR) in an adult population aged ≥ 18 years. This has relevance to long-term consumption of protein on physical function and cardiovascular health in adults, informing dietary choices made by consumers from a lifestyle and sustainability perspective.

Findings from the current meta-analysis showed that no differences were observed in LBM following supplementation of PBP vs. ABP. This evidence is in line with previous reviews which included mainly short-term studies (lasting less than 6 months) (6, 8), and demonstrated similar effects of PBP vs. ABP on LBM. However, the present review finding is in contrast with a recent meta-analysis, which reported that gains in lean mass tended to favor the use of animal protein over plant protein, and their effects were more evident in younger adults (5, 14). The discrepancy may partly be due to the inclusion of mainly short-term trials, which were performed in younger adults (5, 14) in the previous meta-analysis.

Although the TBM estimated in this review was pooled based on heterogeneous populations (healthy and patients), the long-term intake of PBP vs. ABP did not result in significant differences. This finding is consistent with a meta-analysis based on interventional trials (lasting up to 6 months) in patients with hypercholesterolemia which suggested that protein sources did not result in body weight differences (49). Furthermore, this review also indicated that long-term intake of PBP compared with ABP showed no differences on FM. This finding was also similar in subgroup analysis by age, protein dose, exercise, study quality, and length of intervention. However, this finding warrants further investigation, as inclusion of a PBP diet would be expected to result in lower FM than ABP.

Moreover, long-term PBP supplementation did not result in differences in muscle strength and physical performance parameters compared with ABP. This evidence is supported by earlier meta-analyses (5, 6, 8, 14), mainly involving short-term and some long-term studies. The observed similarity in muscle strength and physical performance in the present review may be partly contributed by studies demonstrating similar long-term effects of PBP vs. ABP on LBM and TBM (35, 40, 44). The finding is not unexpected, considering that an increase in body weight positively correlates with increased lean body mass and muscle strength (50), and the latter with physical performance (51).

However, an important factor to consider in evaluating this finding is background dietary intake, which may impact the efficacy of additional protein supplementation. From the studies reporting baseline dietary intake, mean protein intake was ~1.27 g/kg/day (range: 0.89–1.85 g/kg/day). Therefore, in the absence of a significant exercise stimulus (with only 3 of the reported studies including exercise as part of the interventional strategy), it is likely that habitual dietary protein was already at a sufficient level (including consumption of mixed [animal and plant-based protein] meals). As such, the addition of targeted protein intake may not have impacted lean mass or body composition dramatically under such circumstances.

Furthermore, the included trials in this review mainly applied soy protein, which has shown similar efficacy for muscle strength and physical performance as whey protein (6). However, further evidence is needed on long-term (≥ 6 months) effects of other PBP protein sources (such as pea protein) on muscle strength and physical performance parameters with or without physical exercise. This is particularly relevant in light of a recent article highlighting that intake of pea protein can be incorporated into muscle to a similar extent as whey protein, which has important implications relating to muscle protein synthesis (52). Therefore, as long as daily protein intake is sufficient, then interventional responses to PBP or ABP should be similar.

Based on this review, the absence of differences on body composition, muscle strength, and physical performance parameters between PBP and ABP sources could have implications for vegetarians and vegans, those undertaking weight management programs, athletes on a PBP supplement, and those wishing to reduce protein from animal/dairy sources or move to more flexitarian approach.

In terms of cardiometabolic risk factors, the long-term effects of PBP on lipid profiles (TC, LDL, HDL, and TG) did not statistically differ from ABP. Our findings disagree with a preceding review, which suggested that PBP supplementation significantly decreased LDL and non-HDL cholesterol more than animal protein (19). The difference may partly be explained by the inclusion of mainly short-term studies, that is, a median follow-up of 6 weeks, in the compared study, which might blur the long-term effects and differences of the protein sources. However, based on subgroup analyses, PBP supplementation had a statistically significant reduction in TG in trials that had participants aged ≥ 60 years. Since triglycerides are associated with cardiometabolic risk, the PBP source may be clinically relevant for older adults with elevated triglyceride levels. Moreover, polyphenols found in plant food sources may contribute to lower triglyceride levels (53).

Moreover, in the sensitivity analysis, PBP showed a reduction effect, that is, smaller in magnitude, in SBP and DBP compared with ABP. Given that soy protein was included as PBP in this comparison, the finding is substantiated by a meta-analysis performed in postmenopausal women, which showed that soy protein supplementation decreased both SBP and DBP when compared to milk or non-soy protein (54). It has been suggested that the anti-hypertensive effects of soy may be related to isoflavone content and/or amino acid composition (i.e., greater arginine, cysteine, and glycine) of soy when compared to dairy and other ABP sources (55–58). More specifically, the intake of these particular amino acids may facilitate lowered blood pressure by increasing nitric oxide bioavailability, decreasing oxidative stress, and inhibiting angiotensin-converting enzyme (57). Moreover, isoflavones, which act as phytoestrogens in mammals, may influence the endothelial function of blood vessels through biological estrogenic mechanisms (57). It is worth mentioning that the TG and blood pressure findings should not be presented as robust positive effects. TG significance appears only in the ≥60 years subgroup, and the SBP/DBP changes emerge mainly from leave-one-out sensitivity analyses, not from the primary overall analysis. Therefore, such findings should be considered as exploratory in nature rather than conclusive. Furthermore, the review demonstrated that PBP supplementation did not show significant differences in FBG, FBI, and HOMA-IR compared with ABP. However, due to the limited number of trials in this review, further meta-analysis with more clinical studies are needed to confirm the finding.

### Strengths and limitations of the review

The strength of this review lies in its inclusion of only long-term RCTs ranging from 6 to 31 months, aimed at clarifying differences in the studied outcomes over time within a population of adults aged 18 years and older. While many studies have focused on short-term interventions with protein supplementation, understanding the long-term effects of protein supplementation on muscle and cardiometabolic health, as well as the potential variations in outcomes based on the type of protein consumed, is of significance. Subgroup analyses were performed to identify possible differences based on factors such as age, protein dose, exercise, study quality, and length of intervention concerning body composition and lipid profile outcomes, which had a greater number of trials compared to other outcomes in the review. Additionally, the pooled effect was reported along with a prediction interval, enhancing the reliability of estimates for future research. The review was also evaluated using the GRADE approach, which may aid in guiding the development of recommendations and guidelines.

However, we must acknowledge that this meta-analysis also had some limitations. To begin, the review included mainly soy protein (17 out of 18 trials) as the PBP supplementation intervention and dairy proteins (whey, casein, and milk) as the ABP supplementation. The majority of the trials included were primarily conducted with postmenopausal women, and only a few trials involved protein interventions alongside exercise. Additionally, the number of studies reviewed was limited, particularly for FBG, FBI, and HOMA-IR parameters. The analysis did not address subgroup analysis based on health conditions, energy matching, energy restriction/weight-loss context, or comparability of the background diet due to the limited number of trials and inadequate information. Although the review performed subgroup analysis and sensitivity analysis to explain the high heterogeneity, the presence of residual heterogeneity and widened prediction intervals would affect the interpretation of the findings. Therefore, overall findings should be interpreted with caution.

## Conclusion

The results of this review suggest that long-term supplementation of PBP (largely soy protein) compared to ABP (all dairy-based) did not show significant differences in body composition, muscle strength, physical performance, and cardiovascular risk factors in the general adult population. The data support no clear evidence of a difference between protein sources at present, as long as adequate protein intake is maintained over the long term. Findings highlight the presence of high heterogeneity and widened prediction intervals requiring further long-term studies to refine the certainty of the estimate. It is important to note that the majority of long-term RCTs utilized soy protein strategies as PBP, so the results should be interpreted cautiously. Additional research is needed to evaluate the effects of PBP vs. ABP supplementation on the presented parameters in populations with lower habitual protein intake, where supplementation or protein fortification may be beneficial.

## Data Availability

The original contributions presented in the study are included in the article/Supplementary material, further inquiries can be directed to the corresponding authors.

## Footnotes

1
https://www.gradepro.org/
