# Supplementary material for: Long-term effects of plant vs. animal protein supplementation on body composition, muscle strength, physical performance, and cardiometabolic risk factors in adults:a systematic review and meta-analysis of randomized controlled trials
Source: Front Nutr. 2026 Apr 1;13:1813846. doi: 10.3389/fnut.2026.1813846 (PMC13078973; doi:10.3389/fnut.2026.1813846)
Supplement: Supplementary file 1 [file Table_1.docx]

**Long-term effect of Plant versus Animal Protein Supplementation on Body Composition, Muscle Strength, Physical Performance, and Cardiometabolic Risk Factors in Adults: A Systematic Review and Meta-Analysis of Randomized Controlled Trials.**

Supplemental Table 1: Search strategy

| Databases | Search string | Final search date (25/2/2025), |
| --- | --- | --- |
| PubMed | ("animal proteins, dietary"[MeSH Terms] OR "meat proteins"[MeSH Terms] OR "milk proteins"[MeSH Terms] OR "egg proteins, dietary"[MeSH Terms] OR "whey protein"[Title/Abstract] OR "casein"[Title/Abstract] OR ("plant proteins, dietary"[MeSH Terms] OR "vegetable protein" [Title/Abstract] OR "legume*"[Title/Abstract] OR "pulses"[Title/Abstract] OR "soy protein"[Title/Abstract] OR "pea"[Title/Abstract] OR "bean*"[Title/Abstract] OR "lupin*"[Title/Abstract] OR "quinoa"[Title/Abstract])) AND ("body composition"[MeSH Terms] “muscle mass” [Title/Abstract] OR “lean body mass” [Title/Abstract] OR “lean mass” [Title/Abstract] OR"fat-free mass" [Title/Abstract] OR "muscle strength" OR "walking speed"[MeSH Terms] OR "gait"[MeSH Terms] OR "Physical Functional Performance"[MeSH Terms] OR “physical performance” [Title/Abstract] OR “muscle function” [Title/Abstract] OR "lipoproteins"[MeSH Terms] OR "cholesterol"[MeSH Terms] OR "hyperlipidemias"[MeSH Terms] OR "triglycerides"[MeSH Terms] OR "lipid*"[Title/Abstract] OR "hdl"[Title/Abstract] OR "high density lipoprotein*"[Title/Abstract] OR "ldl"[Title/Abstract] OR "low density lipoprotein*"[Title/Abstract] OR “lipid profile*” [Title/Abstract] OR "blood pressure" [Title/Abstract] OR "fasting blood insulin" [Title/Abstract] OR "fasting blood glucose"[Title/Abstract] OR “insulin sensitivity” [Title/Abstract] OR “insulin resistance” [Title/Abstract]) AND "adult*" [Title/Abstract] NOT animal* [Title/Abstract] | 824  (limited to English language publications) |
| Scopus | ( TITLE-ABS-KEY ( "animal protein*" OR "meat proteins" OR "milk proteins" OR "egg proteins" OR "whey protein" OR "casein" ) OR TITLE-ABS-KEY ( "plant protein*" OR "vegetable protein" OR "legume*" OR "pulses" OR "soy protein" OR "pea" OR "bean*" OR "lupin*" OR "quinoa" ) AND TITLE-ABS-KEY ( "body composition" OR "muscle mass" OR "lean body mass" OR "lean mass" OR "fat-free mass" OR "muscle strength" OR "walking speed" OR "gait" OR "Physical Functional Performance" OR "physical performance" OR " muscle function" OR "lipoproteins" OR "cholesterol" OR "hyperlipidemias" OR "triglycerides" OR "lipid*" OR "hdl" OR "high density lipoprotein*" OR "ldl" OR "low density lipoprotein*" OR "lipid profile*" OR "blood pressure" OR "fasting blood insulin" OR "fasting blood glucose" OR "insulin sensitivity" OR "insulin resistance" ) AND TITLE-ABS-KEY ( "adult*" ) not TITLE-ABS-KEY ( "animal*" ) ) | 1695  (limited to English language publications) |
| Web of Science | (((TS=("animal protein*" OR "meat protein" OR "milk protein" OR "egg protein" OR "whey protein" OR "casein" )) OR TS=("plant protein*" OR "vegetable protein" OR "legume*" OR "pulses" OR “soy protein” OR "pea" OR "bean*" OR "lupin*" OR "quinoa")) AND TS=("body composition" OR “muscle mass” OR “lean body mass” OR “ lean mass” OR "fat-free mass" OR "muscle strength" OR "walking speed" OR "gait" OR "physical functional performance" OR “physical performance” OR “muscle function” OR "lipoproteins" OR "cholesterol" OR "hyperlipidemias" OR "triglycerides" OR "lipid*" OR "hdl" OR "high density lipoprotein*" OR "ldl" OR "low density lipoprotein*" OR “lipid profile*” OR "blood pressure" OR "fasting blood insulin" OR "fasting blood glucose" OR “insulin sensitivity” OR “insulin resistance” )) AND TS=("adult*") NOT TS=(animal*) | 1693  (limited to English language publications) |

Supplemental Table 2. Results of sub-group analysis for the long-term effect of plant versus animal protein supplementation on body composition parameters in Adults.

| Outcomes | Sub-group variables | | Number of studies | SMD (95% CI) | P-value for within sub-group heterogenity | I^2^ | P-value for between sub-group heterogenity |
| --- | --- | --- | --- | --- | --- | --- | --- |
| Lean body mass | Age (years) | < 60 | 4 | 0.26(-1.76;2.27) | < 0.0001 | 96.6 | 0.90 |
|  |  | ≥ 60 | 4 | 0.13(-0.19;0.45) | 0.329 | 12.6 |  |
|  | Duration of intervention (months) | < 9 | 5 | 0.38(-1.08;1.83) | < 0.0001 | 94.9 | 0.73 |
|  |  | ≥ 9 | 3 | 0.06(-1.01;1.13) | 0.0024 | 83.5 |  |
|  | Protein dose (g) | < 30 | 5 | -0.17(-0.86;0.53) | 0.0025 | 75.7 | 0.34 |
|  |  | ≥ 40 | 3 | 0.97(-1.31; 3.24) | < 0.0001 | 97 |  |
|  | Exercise | Yes | 3 | -0.17(-1.06;0.72) | 0.0109 | 77.9 | 0.39 |
|  |  | No | 6 | 0.47(-0.70;1.65) | < 0.0001 | 93.5 |  |
|  | Risk of bias | Some concerns | 4 | 0.26(-1.76;2.27) | < 0.0001 | 96.6 | 0.18 |
|  |  | Low | 2 | -0.05(-0.42;0.32) | 0.76 | 0 |  |
|  |  | High | 2 | 0.62(0.01;1.24) | 0.88 | 0 |  |
| Fat mass | Age (years) | < 60 | 4 | 0.18(-1.15;1.52) | < 0.0001 | 92.9 | 0.94 |
|  |  | ≥ 60 | 3 | 0.14(-0.31;0.59) | 0.262 | 25.3 |  |
|  | Duration of intervention (months) | < 9 | 4 | 0.19(-1.14,1.52) | < 0.0001 | 92.9 | 0.92 |
|  |  | ≥ 9 | 3 | 0.12(-0.35;0.59) | 0.272 | 23.1 |  |
|  | Protein dose (g) | < 30 | 4 | -0.27(-1.12;0.57) | 0.007 | 75.3 | 0.17 |
|  |  | ≥ 40 | 3 | 0.70(-0.45; 1.85) | < 0.0001 | 92.2 |  |
|  | Exercise | Yes | 3 | 0.11(-0.26;0.48) | 0.36 | 1.9 | 0.84 |
|  |  | No | 4 | 0.25(-1.11;1.61) | < 0.0001 | 91 |  |
|  | Risk of bias | Some concerns | 4 | 0.18(-1.15;1.52) | < 0.0001 | 92.9 | 0.92 |
|  |  | Low | 1 | 0.25(-0.30;0.80) | - | - |  |
|  |  | High | 2 | 0.03(-0.91;0.96) | 0.12 | 58 |  |
| Total body mass | Age (years) | < 60 | 4 | 0.74(-0.87;2.36) | < 0.0001 | 95.3 | 0.38 |
|  |  | ≥ 60 | 5 | 0.02(-0.16;0.20) | 0.615 | 0 |  |
|  | Duration of intervention (months) | < 9 | 5 | 0.63(-0.62,1.88) | < 0.0001 | 93.8 | 0.33 |
|  |  | ≥ 9 | 4 | 0.01(-0.19;0.20) | 0.377 | 3.1 |  |
|  | Protein dose (g) | < 30 | 6 | -0.00(-0.18;0.17) | 0.649 | 0 | 0.31 |
|  |  | ≥ 40 | 3 | 1.07(-1.02 ; 3.16) | < 0.0001 | 96.6 |  |
|  | Exercise | Yes | 3 | -0.09(-0.46;0.28) | 0.71 | 0.0 | 0.20 |
|  |  | No | 6 | 0.62(-0.41;1.65) | < 0.0001 | 92.8 |  |
|  | Risk of bias | Some concerns | 4 | 0.74(-0.87;2.36) | < 0.0001 | 95.3 | 0.58 |
|  |  | Low | 3 | 0.00(-0.19;0.19) | 0.87 | 0 |  |
|  |  | High | 2 | 0.24(-0.61;1.08) | 0.16 | 48 |  |

Supplemental Table 3. Results of sub-group analysis for the long-term effect of plant versus animal protein supplementation on lipid profile parameters in Adults.

| Outcomes | Sub-group | | Number of studies | SMD (95% CI) | P-value for within sub-group heterogenity | I^2^ | P-value for between sub-group heterogenity |
| --- | --- | --- | --- | --- | --- | --- | --- |
| TC | Age (years) | < 60 | 4 | -0.17(-0.56;0.23) | 0.036 | 64.8 | 0.43 |
|  |  | ≥ 60 | 3 | -0.66(-1.83;0.51) | < 0.0001 | 93.9 |  |
|  | Duration of intervention (months) | < 9 | 4 | -0.44(-1.35;0.48) | < 0.0001 | 90.9 | 0.78 |
|  |  | ≥ 9 | 3 | -0.29(-0.77;0.18) | 0.0105 | 78.1 |  |
|  | Protein dose (g) | < 30 | 4 | -0.20(-0.55;0.15) | 0.013 | 71.9 | 0.52 |
|  |  | ≥ 30 | 3 | -0.61(-1.84;0.62) | < 0.0001 | 93.1 |  |
|  | Risk of bias | Some concerns | 4 | -0.66(-1.54;0.22) | < 0.0001 | 90.1 | 0.19 |
|  |  | Low | 3 | -0.07(-0.30;0.16) | 0.15 | 47.2 |  |
| LDL | Age (years) | < 60 | 4 | -0.08(-0.33;0.16) | 0.27 | 23.2 | 0.37 |
|  |  | ≥ 60 | 3 | -0.68(-1.97;0.61) | < 0.0001 | 94.8 |  |
|  | Duration of intervention (months) | < 9 | 4 | -0.48 (-1.46,0.50) | < 0.0001 | 91.8 | 0.55 |
|  |  | ≥ 9 | 3 | -0.16(-0.51; 0.18) | 0.036 | 69.7 |  |
|  | Protein dose (g) | < 30 | 4 | -0.12(-0.37;0.13) | 0.067 | 58 | 0.44 |
|  |  | ≥ 40 | 3 | -0.65(-1.99;0.68) | < 0.0001 | 94 |  |
|  | Risk of bias | Some concerns | 4 | -0.61(-1.55;0.33) | < 0.0001 | 91 | 0.26 |
|  |  | Low | 3 | -0.05(-0.30;0.20) | 0.09 | 58.4 |  |
| HDL | Age (years) | < 60 | 4 | -0.11(-0.46; 0.24) | 0.05 | 59.8 | 0.89 |
|  |  | ≥ 60 | 3 | -0.01(-1.53;1.52) | < 0.0001 | 97.8 |  |
|  | Duration of intervention (months) | < 9 | 4 | 0.29(-0.37;0.94) | 0.0002 | 85.1 | 0.14 |
|  |  | ≥ 9 | 3 | -0.56(-1.52;0.39) | < 0.0001 | 96.9 |  |
|  | Protein dose (g) | < 30 | 4 | -0.36(-1.14;0.43) | < 0.0001 | 95.9 | 0.29 |
|  |  | ≥ 40 | 3 | 0.30(-0.64;1.25) | < 0.0001 | 90 |  |
|  | Risk of bias | Some concerns | 4 | 0.11(-0.65;0.87) | < 0.0001 | 88.1 | 0.52 |
|  |  | Low | 3 | -0.33(-1.41;0.76) | < 0.0001 | 97.2 |  |
| TG | Age (years) | < 60 | 4 | 0.11(-0.14; 0.36) | 0.26 | 23.7 | 0.007 |
|  |  | ≥ 60 | 3 | -0.39(-0.65;-0.13) | 0.133 | 50.4 |  |
|  | Duration of intervention (months) | < 9 | 4 | -0.09(-0.57;0.39) | 0.006 | 75.9 | 0.54 |
|  |  | ≥ 9 | 3 | -0.26(-0.5; - 0.01) | 0.17 | 43.2 |  |
|  | Protein dose (g) | < 30 | 4 | -0.12(-0.42;0.17) | 0.02 | 63.5 | 0.80 |
|  |  | ≥ 40 | 3 | -0.21(-0.84;0.42) | 0.008 | 79.4 |  |
|  | Risk of bias | Some concerns | 4 | -0.15(-0.61;0.30) | 0.018 | 70.1 | 0.97 |
|  |  | Low | 3 | -0.14(-0.51;0.22) | 0.0092 | 78.7 |  |

Supplemental Table 4. Sensitivity analysis of the systematic removal of each trial for the long-term effect of plant versus animal protein supplementation on body composition (lean body mass, fat mass, total body mass).

| Outcome | Omitted study | Estimate | P -value | 95% confidence interval of the estimate | | Q | Q, p-value | tau^2^ | I^2^ | H^2^ |
| --- | --- | --- | --- | --- | --- | --- | --- | --- | --- | --- |
|  |  |  |  | Lower | Upper |  |  |  |  |  |
| Lean body mass | Moeller et al., 2003 | -0.18 | 0.40 | -0.60 | 0.24 | 15.84 | 0.01 | 0.20 | 66.44 | 2.98 |
|  | Evans et al., 2007 (I) | 0.21 | 0.70 | -0.85 | 1.28 | 89.47 | 0.00 | 1.95 | 95.14 | 20.59 |
|  | Evans et al., 2007 (II) | 0.22 | 0.68 | -0.85 | 1.29 | 89.84 | 0.00 | 1.96 | 95.15 | 20.62 |
|  | Volek et al., 2013 | 0.44 | 0.39 | -0.57 | 1.44 | 79.36 | 0.00 | 1.70 | 94.10 | 16.95 |
|  | Tomayko et al., 2014 | 0.44 | 0.39 | -0.56 | 1.43 | 84.07 | 0.00 | 1.69 | 94.43 | 17.95 |
|  | Kjølbæk et al., 2017 | 0.34 | 0.53 | -0.73 | 1.41 | 87.66 | 0.00 | 1.94 | 94.09 | 16.92 |
|  | Li et al., 2021 | 0.30 | 0.58 | -0.78 | 1.38 | 90.74 | 0.00 | 1.98 | 94.41 | 17.89 |
|  | Jadczak et al., 2021 | 0.32 | 0.56 | -0.76 | 1.39 | 90.25 | 0.00 | 1.96 | 94.58 | 18.44 |
| Fat mass | Moeller et al., 2003 | -0.10 | 0.69 | -0.57 | 0.38 | 13.01 | 0.02 | 0.23 | 67.02 | 3.03 |
|  | Evans et al., 2007 (I) | 0.26 | 0.54 | -0.57 | 1.08 | 41.92 | 0.00 | 0.94 | 90.23 | 10.23 |
|  | Evans et al., 2007 (II) | 0.11 | 0.80 | -0.74 | 0.96 | 44.66 | 0.00 | 1.00 | 90.83 | 10.90 |
|  | Volek et al., 2013 | 0.15 | 0.73 | -0.71 | 1.02 | 44.84 | 0.00 | 1.04 | 90.25 | 10.25 |
|  | Tomayko et al., 2014 | 0.41 | 0.20 | -0.22 | 1.04 | 30.23 | 0.00 | 0.51 | 83.59 | 6.09 |
|  | Kjølbæk et al., 2017 | 0.19 | 0.67 | -0.68 | 1.06 | 42.93 | 0.00 | 1.04 | 88.99 | 9.09 |
|  | Jadczak e tal., 2021 | 0.15 | 0.74 | -0.72 | 1.01 | 44.86 | 0.00 | 1.04 | 89.86 | 9.86 |
| Total Body mass | Moeller et al., 2003 | -0.004 | 0.964 | -0.162 | 0.155 | 3.252 | 0.861 | 0.000 | 0.000 | 1.000 |
|  | Evans et al., 2007 (I) | 0.431 | 0.273 | -0.340 | 1.202 | 71.424 | 0.000 | 1.137 | 94.567 | 18.405 |
|  | Evans et al., 2007 (II) | 0.335 | 0.399 | -0.444 | 1.113 | 70.626 | 0.000 | 1.164 | 94.711 | 18.908 |
|  | Hodis et al., 2011 | 0.416 | 0.302 | -0.374 | 1.207 | 69.471 | 0.000 | 1.178 | 91.941 | 12.408 |
|  | Volek et al., 2013 | 0.445 | 0.258 | -0.326 | 1.216 | 70.393 | 0.000 | 1.128 | 94.235 | 17.345 |
|  | Tomayko et al., 2014 | 0.401 | 0.315 | -0.380 | 1.182 | 71.968 | 0.000 | 1.169 | 94.690 | 18.834 |
|  | Kjølbæk et al., 2017 | 0.423 | 0.291 | -0.361 | 1.207 | 71.219 | 0.000 | 1.165 | 93.847 | 16.251 |
|  | Li et al.,2021 | 0.429 | 0.281 | -0.352 | 1.210 | 70.956 | 0.000 | 1.155 | 94.018 | 16.717 |
|  | Jadczak e tal., 2021 | 0.405 | 0.313 | -0.382 | 1.193 | 71.929 | 0.000 | 1.178 | 94.308 | 17.568 |

Supplemental Table 5. Sensitivity analysis of the systematic removal of each trial for the long-term effect of plant versus animal protein supplementation on muscle strength and physical performance.

| Outcome | Omitted study | Estimate | P -value | 95% confidence interval of the estimate | | Q | Q, p-value | tau^2^ | I^2^ | H^2^ |
| --- | --- | --- | --- | --- | --- | --- | --- | --- | --- | --- |
|  |  |  |  | Lower | Upper |  |  |  |  |  |
| Upper body muscle strength | Vupadhyayula et al., 2009 | -0.7 | 0.4 | -2.1 | 0.8 | 26.5 | 0.0 | 1.5 | 94.4 | 17.7 |
|  | Li et al., 2021 | -1.2 | 0.1 | -2.5 | 0.1 | 27.1 | 0.0 | 1.2 | 93.8 | 16.1 |
|  | Jadczak et al., 2021 | -1.2 | 0.1 | -2.5 | 0.1 | 33.2 | 0.0 | 1.3 | 94.7 | 18.9 |
|  | Volek et al., 2013(I) | -0.5 | 0.4 | -1.5 | 0.5 | 32.4 | 0.0 | 0.7 | 92.8 | 13.8 |
| Lower body muscle strength | Vupadhyayula et al., 2009 | 0.64 | 0.14 | -0.21 | 1.49 | 2.55 | 0.11 | 0.23 | 60.76 | 2.55 |
|  | Tomayko et al., 2014 | 3.20 | 0.28 | -2.57 | 8.97 | 139.82 | 0.00 | 17.22 | 99.28 | 139.82 |
|  | Volek et al., 2013(II) | 3.65 | 0.15 | -1.27 | 8.56 | 71.51 | 0.00 | 12.39 | 98.60 | 71.51 |
| Timed Up and Go (TUG) | Vupadhyayula et al., 2009 | -0.22 | 0.35 | -0.68 | 0.24 | 0.13 | 0.72 | 0.00 | 0.00 | 1.00 |
|  | Tomayko et al., 2014 | -1.01 | 0.16 | -2.42 | 0.40 | 17.67 | 0.00 | 0.98 | 94.34 | 17.67 |
|  | Jadczak et al., 2021 | -0.95 | 0.24 | -2.53 | 0.64 | 12.35 | 0.00 | 1.21 | 91.90 | 12.35 |
| Chair stand test | Li et al.,2021 | 1.64 | 0.00 | 0.70 | 2.59 | 0.00 | 1.00 | 0.00 | 0.00 | 1.00 |
|  | Tomayko et al., 2014 | -0.10 | 0.70 | -0.60 | 0.40 | 0.00 | 1.00 | 0.00 | 0.00 | 1.00 |
| SPPB | Kok et al.,2005 | -0.37 | 0.32 | -1.09 | 0.36 | 3.35 | 0.07 | 0.19 | 70.16 | 3.35 |
|  | Li et al., 2021 | 0.00 | 1.00 | -0.27 | 0.27 | 0.00 | 1.00 | 0.00 | 0.00 | 1.00 |
|  | Jadczak et al., 2021 | -0.33 | 0.37 | -1.05 | 0.39 | 4.97 | 0.03 | 0.22 | 79.88 | 4.97 |

Supplemental Table 6. Sensitivity analysis of the systematic removal of each trial for the long-term effect of plant versus animal protein supplementation on lipid profile parameters.

| Outcome | Omitted study | Estimate | P -value | 95% confidence interval of the estimate | | Q | Q, p-value | tau^2^ | I^2^ | H^2^ |
| --- | --- | --- | --- | --- | --- | --- | --- | --- | --- | --- |
|  |  |  |  | Lower | Upper |  |  |  |  |  |
| TC | Baum et al.,1998 | -0.13 | 0.27 | -0.35 | 0.10 | 11.33 | 0.05 | 0.04 | 55.81 | 2.26 |
|  | Dent et al., 2001 | -0.43 | 0.15 | -1.02 | 0.15 | 41.08 | 0.00 | 0.48 | 93.38 | 15.10 |
|  | Kreijkamp-Kaspers et al., 2004 | -0.45 | 0.12 | -1.03 | 0.12 | 36.99 | 0.00 | 0.47 | 91.69 | 12.04 |
|  | Campbell et al.,2010 | -0.30 | 0.30 | -0.87 | 0.27 | 36.30 | 0.00 | 0.46 | 93.08 | 14.45 |
|  | Hodis et al., 2011 | -0.40 | 0.20 | -1.01 | 0.21 | 41.64 | 0.00 | 0.52 | 91.51 | 11.78 |
|  | Liu et al., 2012 | -0.45 | 0.13 | -1.03 | 0.13 | 39.31 | 0.00 | 0.47 | 92.49 | 13.32 |
|  | Kjølbæk et al., 2017 | -0.44 | 0.14 | -1.02 | 0.14 | 40.52 | 0.00 | 0.48 | 93.10 | 14.50 |
| LDL | Baum et al.,1998 | -0.08 | 0.41 | -0.27 | 0.11 | 8.36 | 0.14 | 0.02 | 39.49 | 1.65 |
|  | Dent et al., 2001 | -0.38 | 0.23 | -1.00 | 0.24 | 43.53 | 0.00 | 0.56 | 94.19 | 17.22 |
|  | Kreijkamp-Kaspers et al., 2004 | -0.43 | 0.16 | -1.03 | 0.17 | 37.24 | 0.00 | 0.51 | 92.34 | 13.05 |
|  | Campbell et al.,2010 | -0.32 | 0.32 | -0.94 | 0.30 | 42.09 | 0.00 | 0.56 | 94.21 | 17.27 |
|  | Hodis et al., 2011 | -0.36 | 0.27 | -1.00 | 0.28 | 43.11 | 0.00 | 0.57 | 92.27 | 12.94 |
|  | Liu et al., 2012 | -0.40 | 0.20 | -1.02 | 0.22 | 42.35 | 0.00 | 0.54 | 93.39 | 15.13 |
|  | Kjølbæk et al., 2017 | -0.42 | 0.17 | -1.02 | 0.17 | 41.16 | 0.00 | 0.51 | 93.51 | 15.42 |
| HDL | Baum et al.,1998 | -0.29 | 0.28 | -0.81 | 0.23 | 73.93 | 0.00 | 0.38 | 91.64 | 11.96 |
|  | Dent et al., 2001 | -0.10 | 0.77 | -0.81 | 0.60 | 97.98 | 0.00 | 0.74 | 95.41 | 21.78 |
|  | Kreijkamp-Kaspers et al., 2004 | 0.15 | 0.53 | -0.31 | 0.60 | 25.91 | 0.00 | 0.27 | 86.86 | 7.61 |
|  | Campbell et al.,2010 | -0.02 | 0.95 | -0.72 | 0.68 | 96.63 | 0.00 | 0.71 | 95.25 | 21.03 |
|  | Hodis et al., 2011 | -0.13 | 0.72 | -0.84 | 0.58 | 85.80 | 0.00 | 0.73 | 93.64 | 15.71 |
|  | Liu et al., 2012 | -0.14 | 0.70 | -0.84 | 0.56 | 93.57 | 0.00 | 0.72 | 94.75 | 19.04 |
|  | Kjølbæk et al., 2017 | -0.03 | 0.93 | -0.74 | 0.67 | 97.22 | 0.00 | 0.73 | 95.21 | 20.88 |
| TG | Baum et al.,1998 | -0.07 | 0.57 | -0.33 | 0.18 | 15.32 | 0.01 | 0.06 | 65.15 | 2.87 |
|  | Dent et al., 2001 | -0.13 | 0.42 | -0.43 | 0.18 | 19.43 | 0.00 | 0.10 | 75.69 | 4.11 |
|  | Kreijkamp-Kaspers et al., 2004 | -0.14 | 0.39 | -0.46 | 0.18 | 19.49 | 0.00 | 0.12 | 73.79 | 3.82 |
|  | Campbell et al.,2010 | -0.17 | 0.29 | -0.47 | 0.14 | 18.94 | 0.00 | 0.10 | 75.52 | 4.09 |
|  | Hodis et al., 2011 | -0.08 | 0.59 | -0.37 | 0.21 | 12.85 | 0.02 | 0.08 | 63.20 | 2.72 |
|  | Liu et al., 2012 | -0.21 | 0.12 | -0.49 | 0.06 | 13.62 | 0.02 | 0.07 | 65.59 | 2.91 |
|  | Kjølbæk et al., 2017 | -0.22 | 0.09 | -0.47 | 0.03 | 13.54 | 0.02 | 0.06 | 63.37 | 2.73 |

Supplemental Table 7. Sensitivity analysis of the systematic removal of each trial for the long-term effect of plant versus animal protein supplementation on blood pressure, FBG, FBI and HOMA-IR.

| Outcomes | Omitted study | Estimate | P -value | 95% confidence interval of the estimate | | Q | Q, p-value | tau^2^ | I^2^ | H^2^ |
| --- | --- | --- | --- | --- | --- | --- | --- | --- | --- | --- |
|  |  |  |  | Lower | Upper |  |  |  |  |  |
| SBP | Kreijkamp-Kaspers et al., 2005 | -0.24 | 0.01 | -0.42 | -0.07 | 0.44 | 0.80 | 0.00 | 0.00 | 1.00 |
|  | Hodis et al.,2011 | -0.11 | 0.58 | -0.50 | 0.28 | 7.16 | 0.03 | 0.08 | 70.00 | 3.33 |
|  | Liu et al., 2013 | -0.07 | 0.66 | -0.40 | 0.25 | 6.53 | 0.04 | 0.06 | 69.09 | 3.23 |
|  | Kjølbæk et al., 2017 | -0.09 | 0.58 | -0.42 | 0.24 | 7.38 | 0.02 | 0.06 | 74.12 | 3.86 |
| DBP | Kreijkamp-Kaspers et al., 2005 | -0.20 | 0.02 | -0.37 | -0.03 | 0.22 | 0.90 | 0.00 | 0.00 | 1.00 |
|  | Hodis et al.,2011 | -0.01 | 0.93 | -0.24 | 0.22 | 2.13 | 0.35 | 0.01 | 16.67 | 1.20 |
|  | Liu et al., 2013 | -0.08 | 0.55 | -0.34 | 0.18 | 4.33 | 0.11 | 0.03 | 53.50 | 2.15 |
|  | Kjølbæk et al., 2017 | -0.08 | 0.50 | -0.33 | 0.16 | 4.33 | 0.11 | 0.02 | 53.46 | 2.15 |
| FBG | Liu et al.,2010 | 0.33 | 0.47 | -0.56 | 1.21 | 11.90 | 0.00 | 0.37 | 91.60 | 11.90 |
|  | Hodis et al., 2011 | -0.04 | 0.80 | -0.32 | 0.24 | 0.36 | 0.55 | 0.00 | 0.00 | 1.00 |
|  | Kjølbæk et al., 2017 | 0.41 | 0.26 | -0.30 | 1.12 | 10.94 | 0.00 | 0.24 | 90.86 | 10.94 |
| FBI | Liu et al., 2010 | 0.03 | 0.91 | -0.43 | 0.49 | 0.00 | 1.00 | 0.00 | 0.00 | 1.00 |
|  | Kjølbæk et al., 2017 | 0.18 | 0.34 | -0.18 | 0.53 | 0.00 | 1.00 | 0.00 | 0.00 | 1.00 |
| HOMA-IR | Liu et al., 2010 | 0.06 | 0.81 | -0.41 | 0.52 | 0.00 | 1.00 | 0.00 | 0.00 | 1.00 |
|  | Kjølbæk et al., 2017 | 0.25 | 0.17 | -0.11 | 0.61 | 0.00 | 1.00 | 0.00 | 0.00 | 1.00 |

A: Lean body mass B: Fat mass C: Total body mass


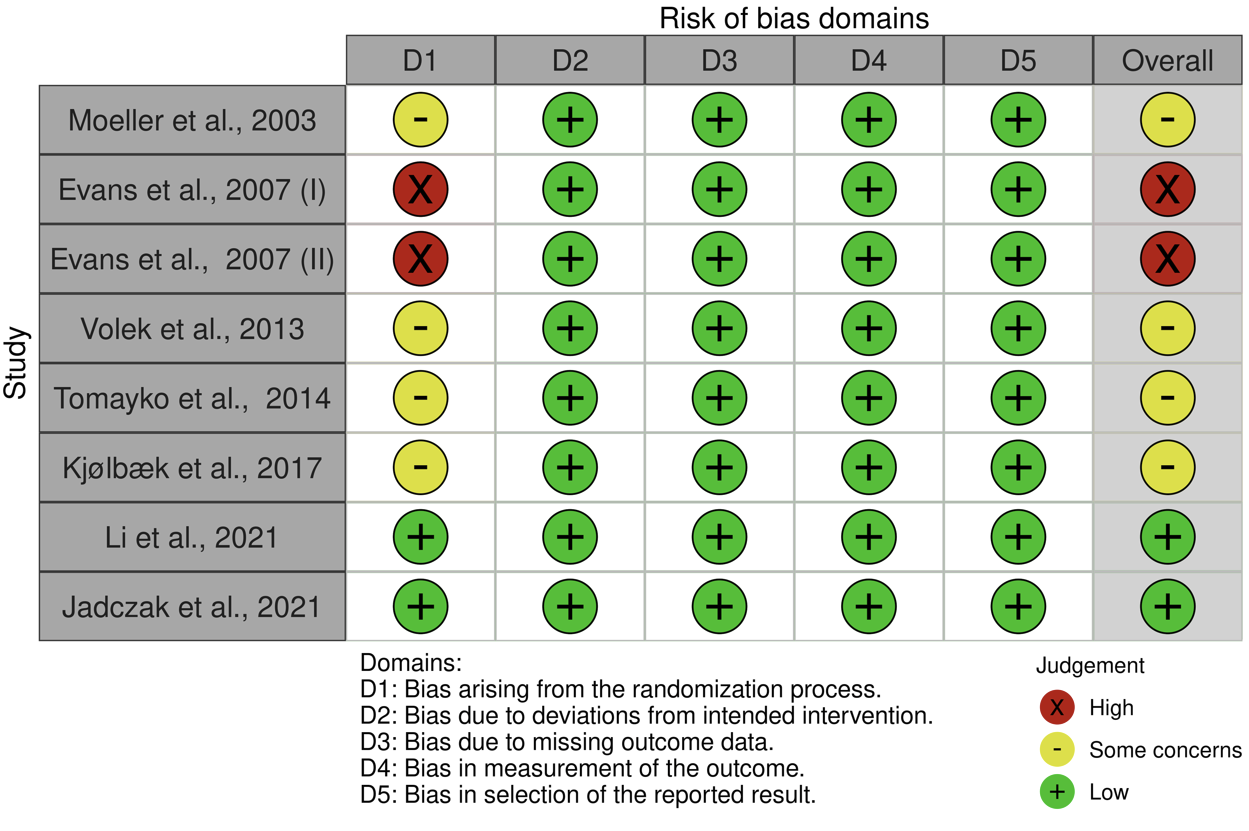

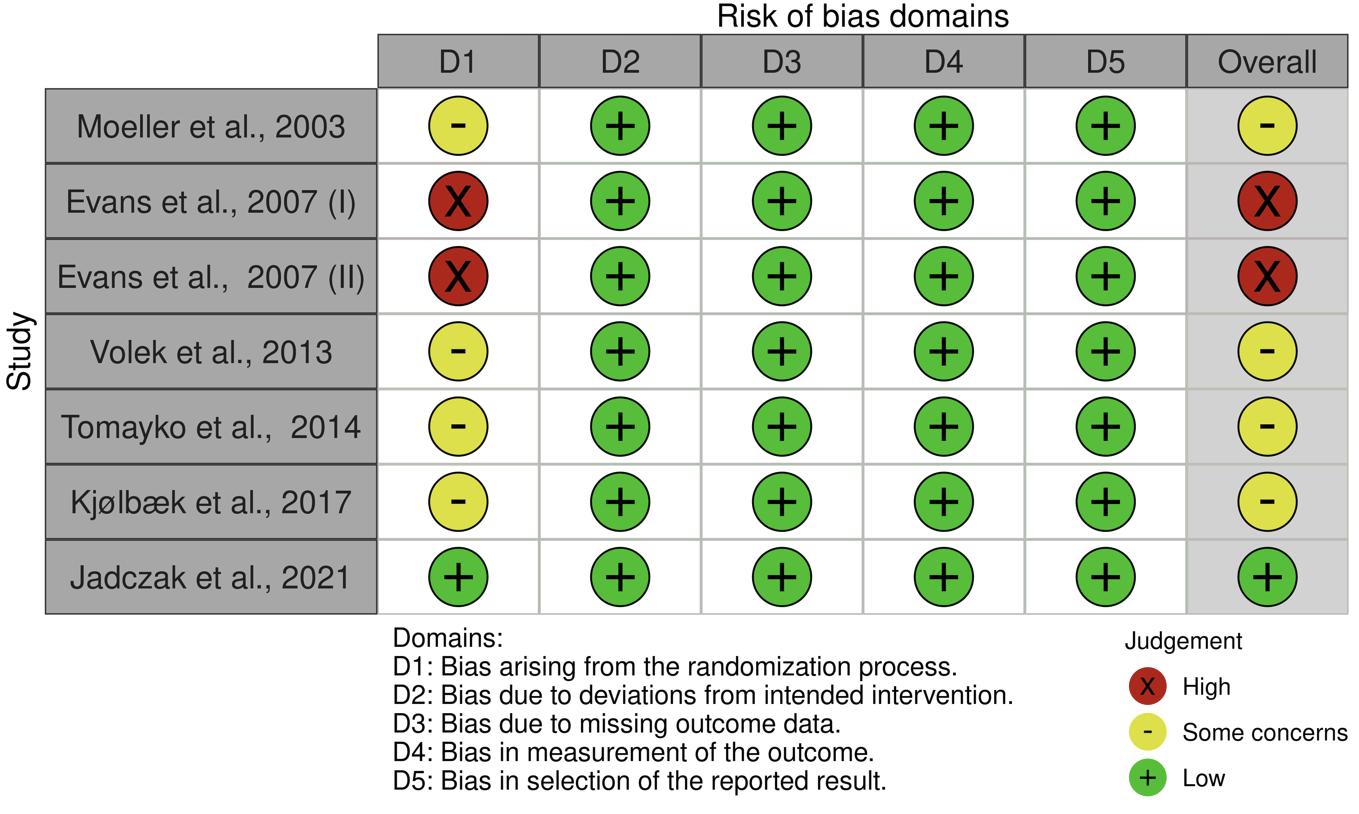

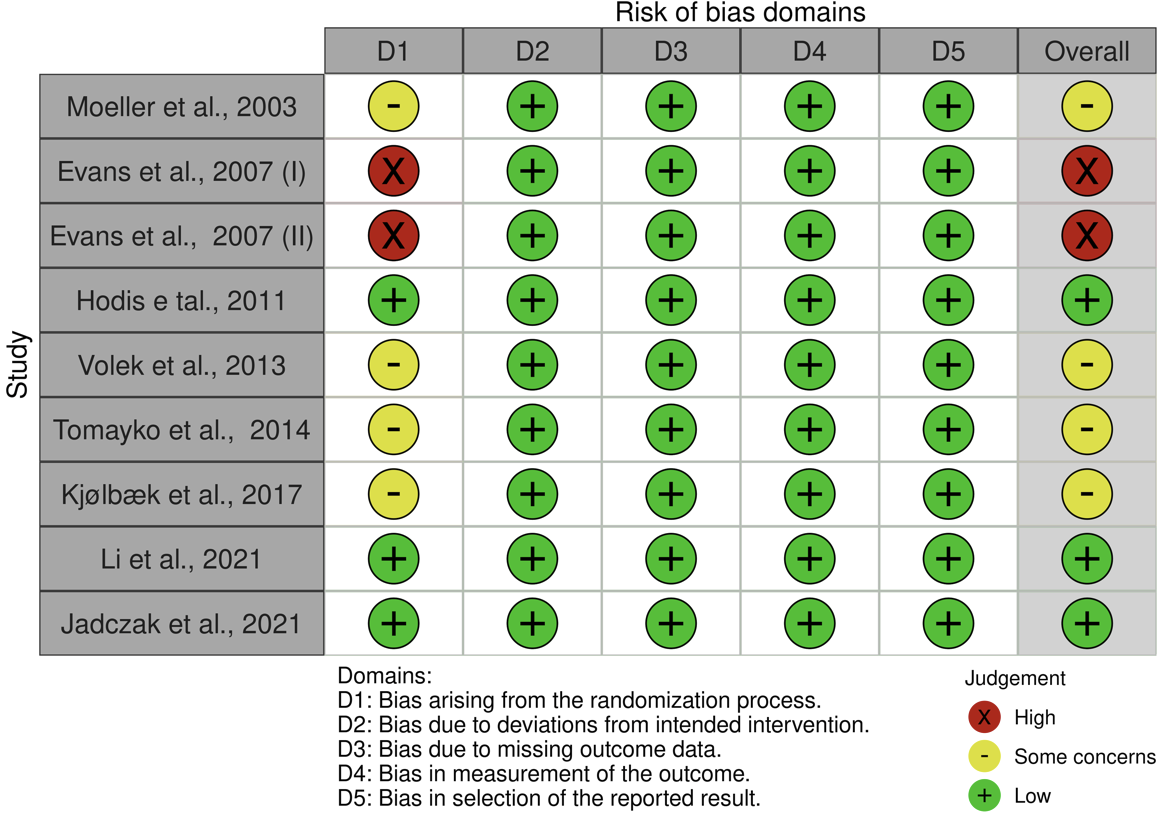


D: Upper body muscle strength E: Lower body muscle strength


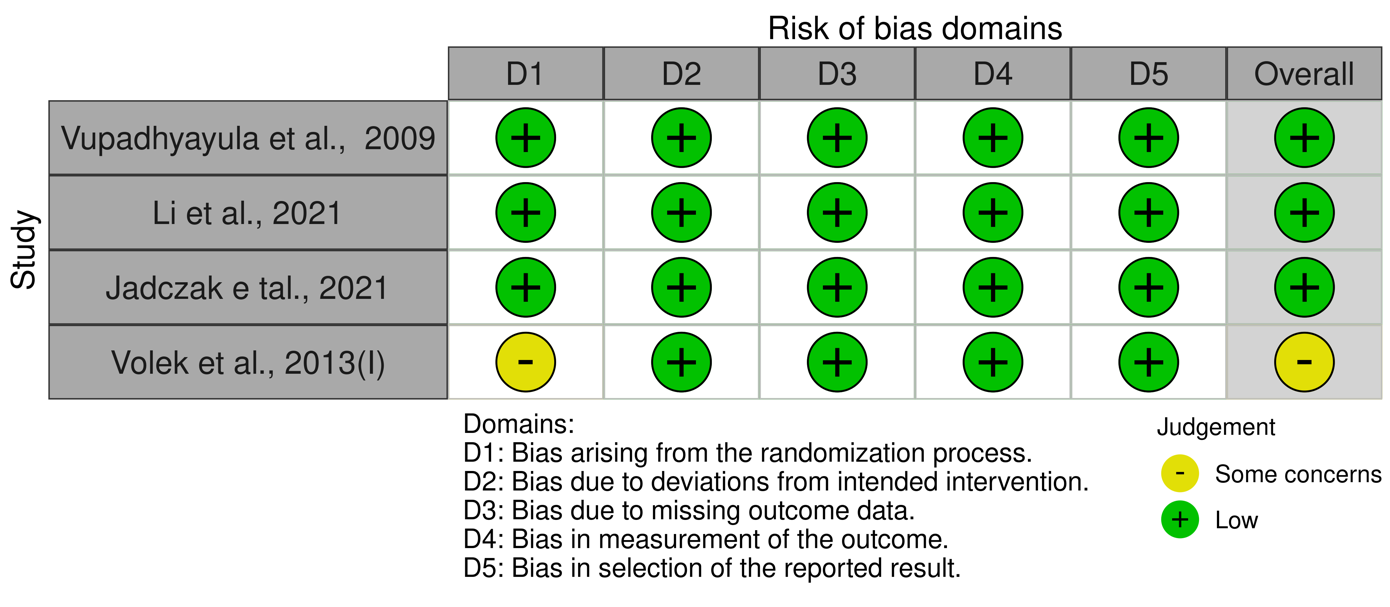

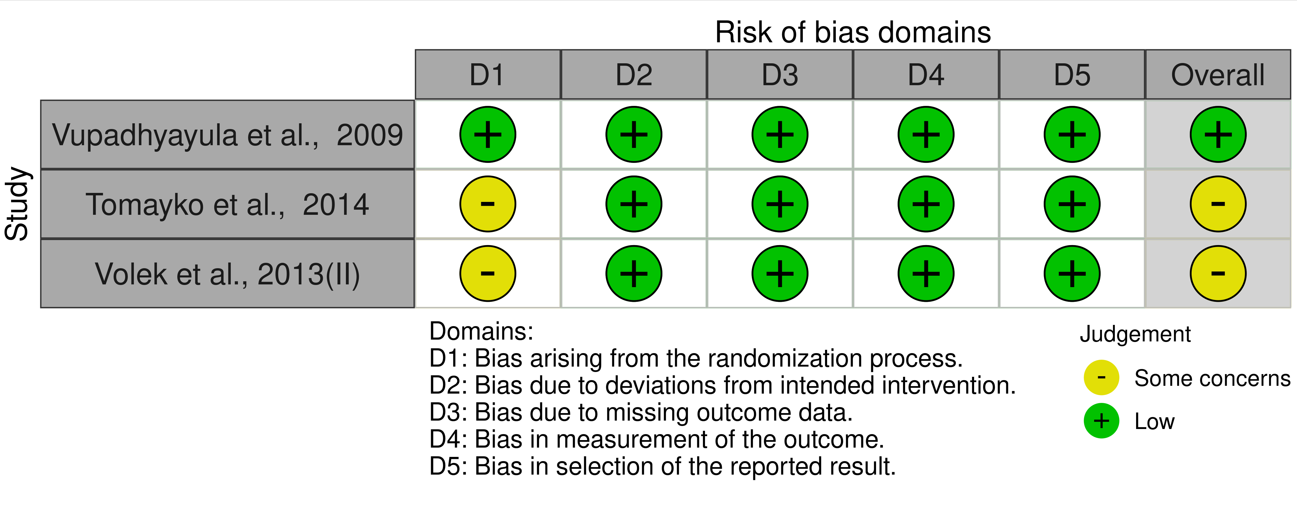


F: Gait speed G: Chair stand test


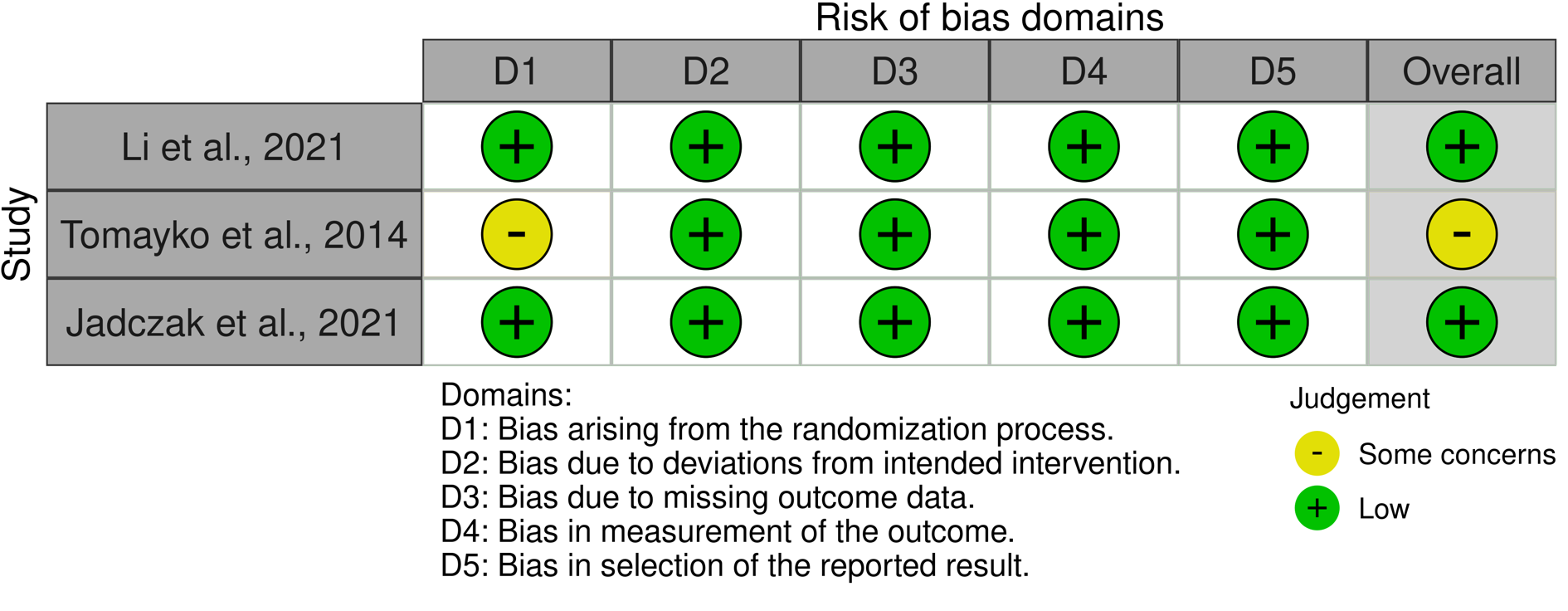

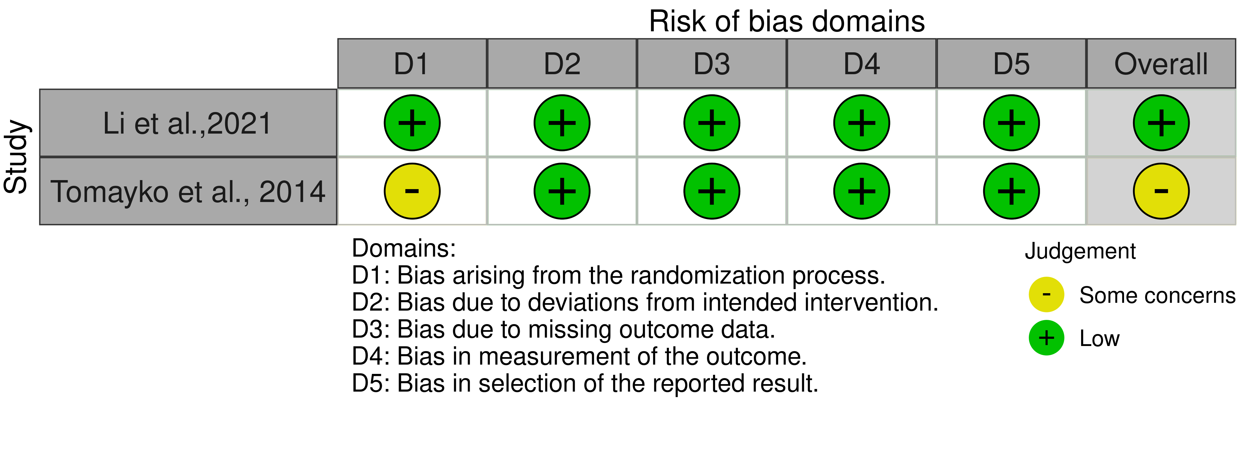


H: Timed Up and Go I: Short physical performance battery


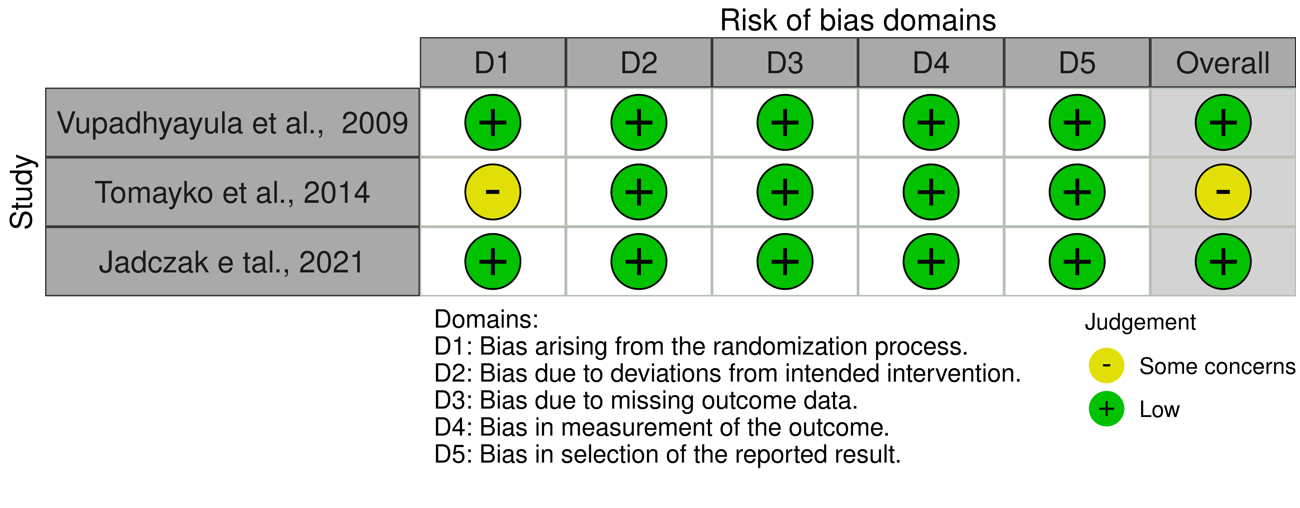

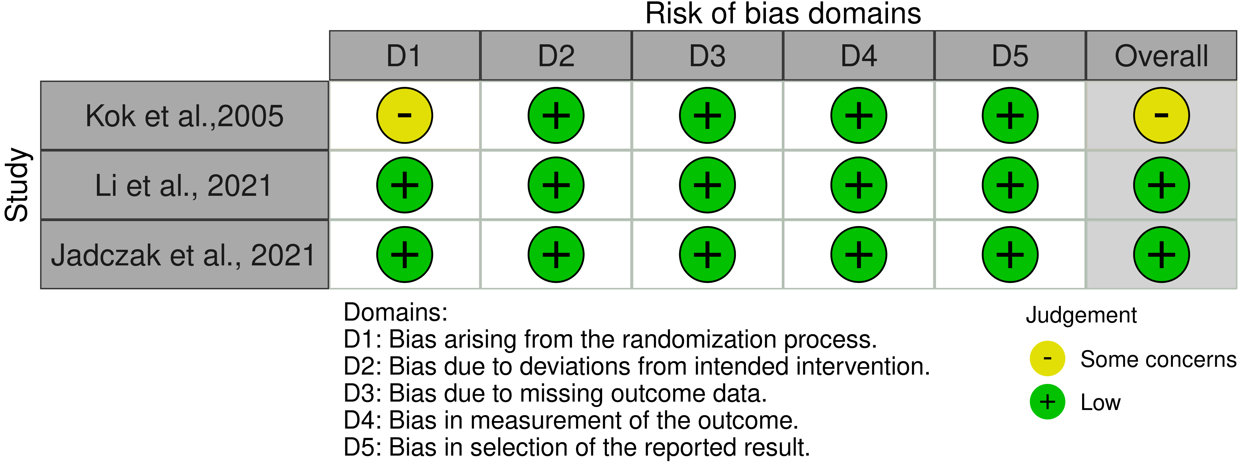


J: lipid profile (TC, LDL, HDL, TG) K: Blood pressure (SBP, DBP)


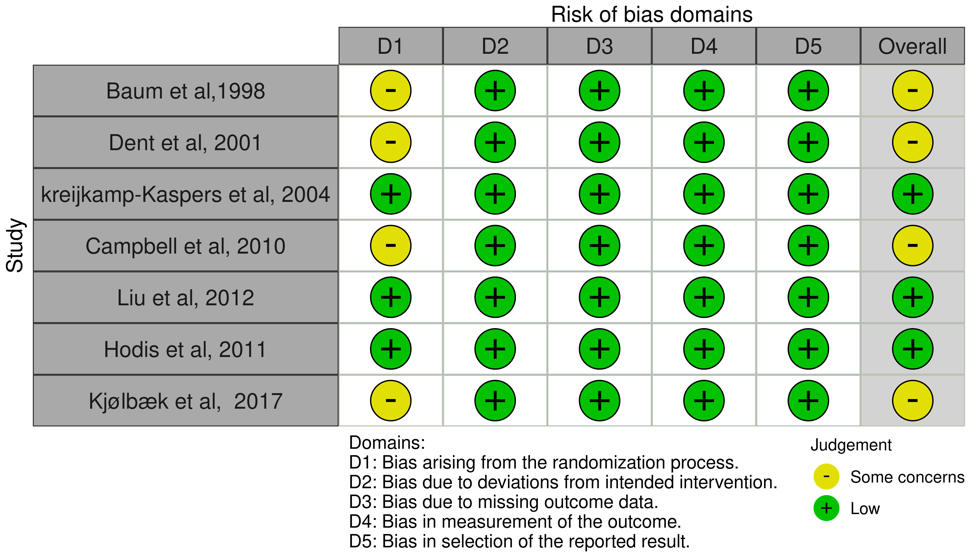

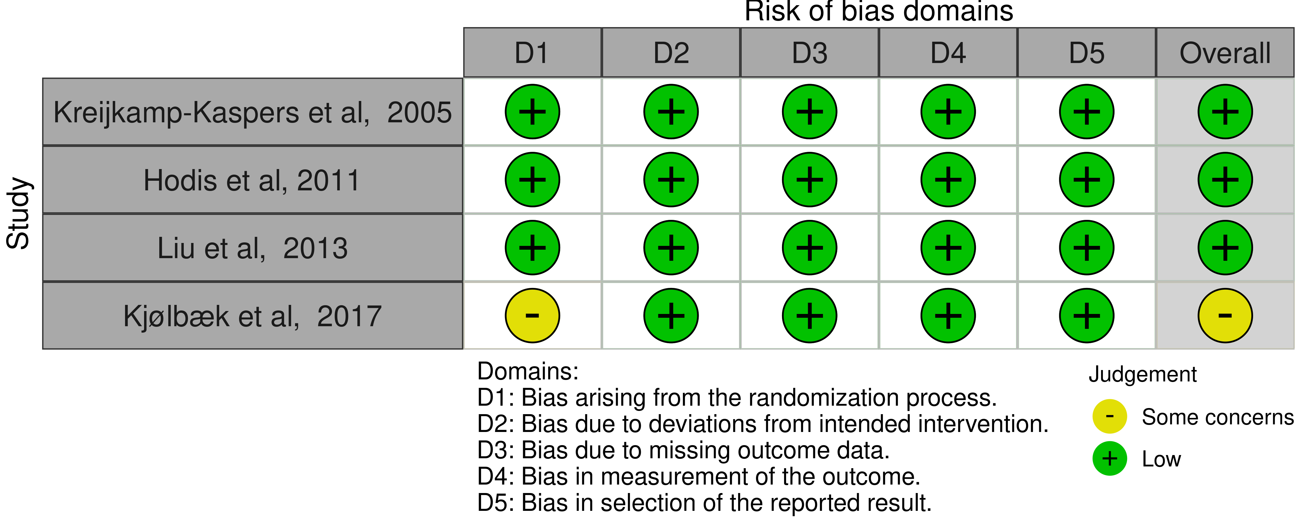


L: Fasting blood glucose (FBG) M: Fasting blood insulin (FBI), and HOMA-IR


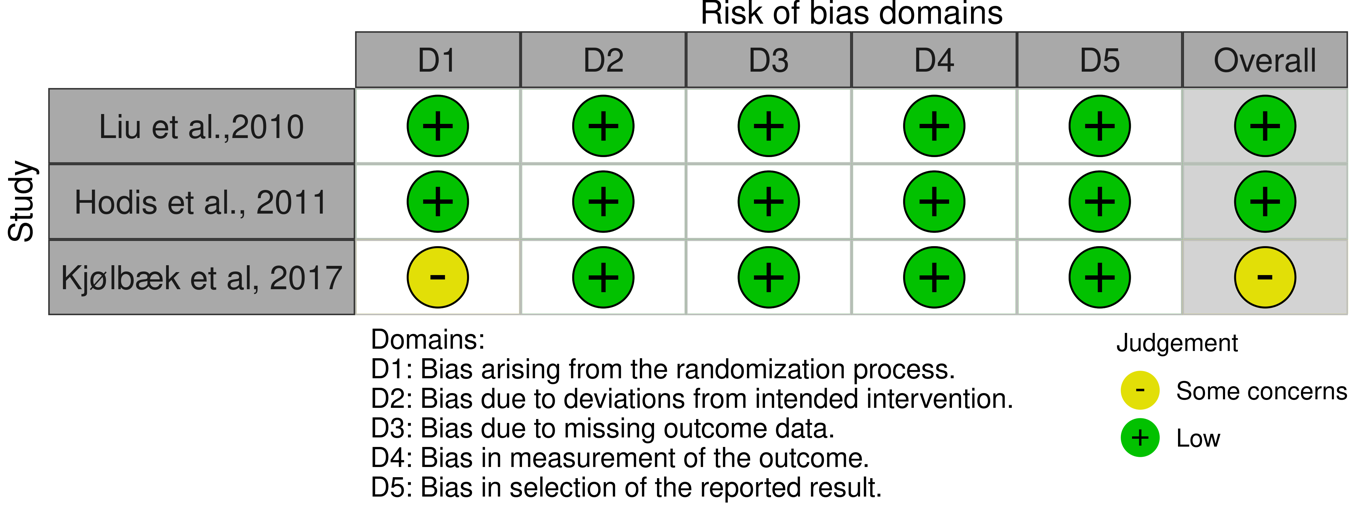

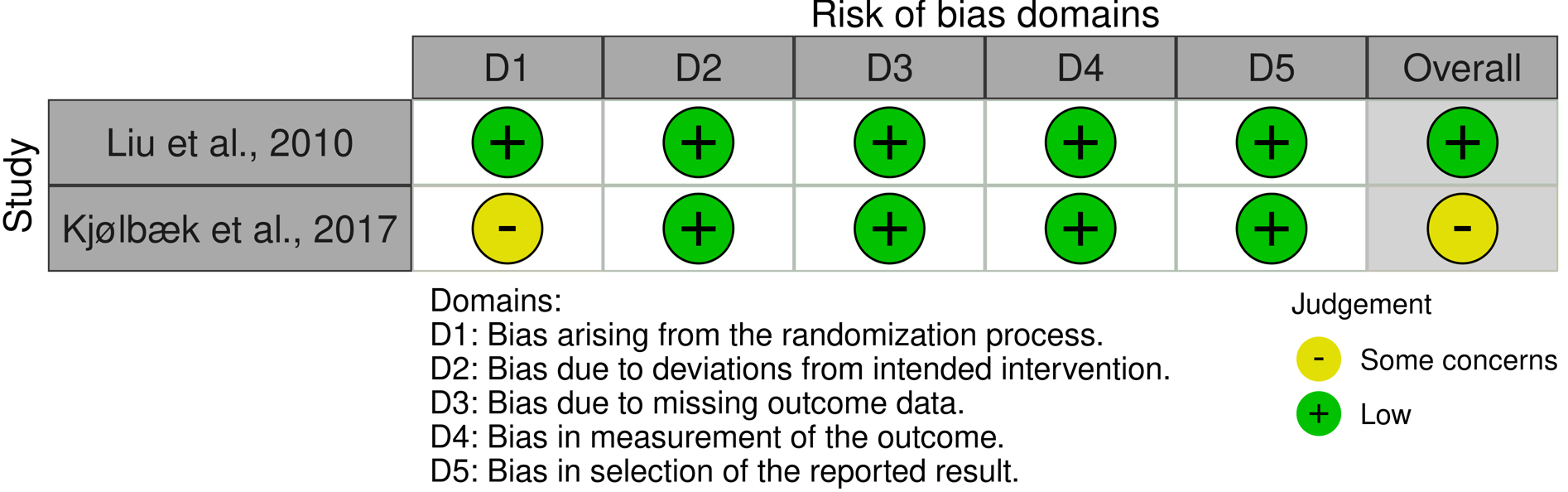


Supplemental Figure 1. Risk of bias assessment results using Rob2 tools for outcomes included in the meta-analysis, From Panel A to M.


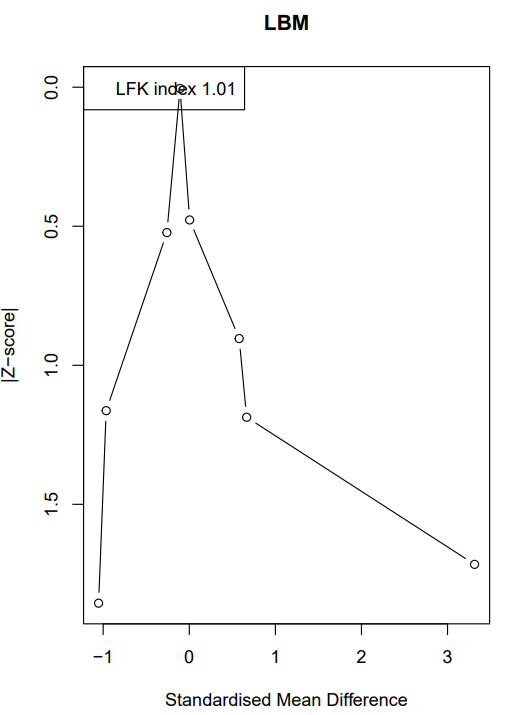

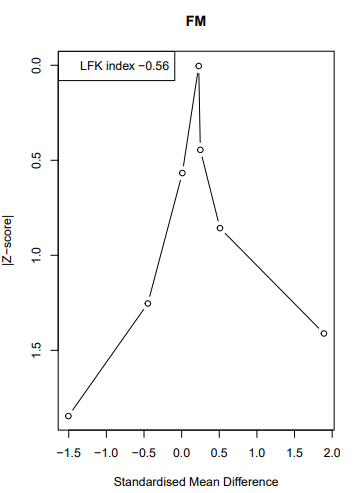

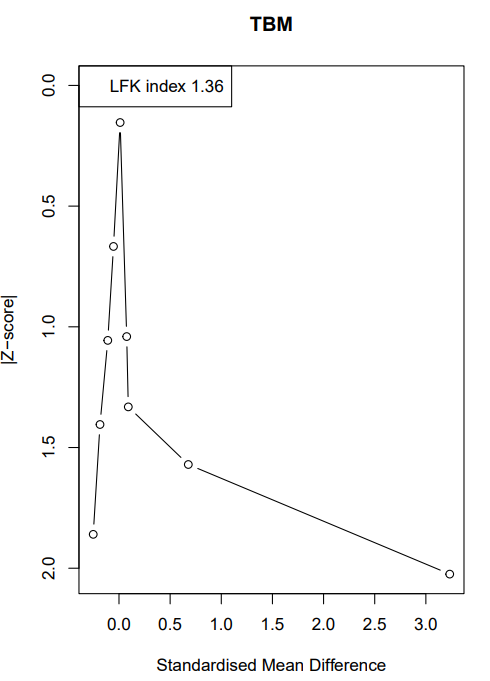


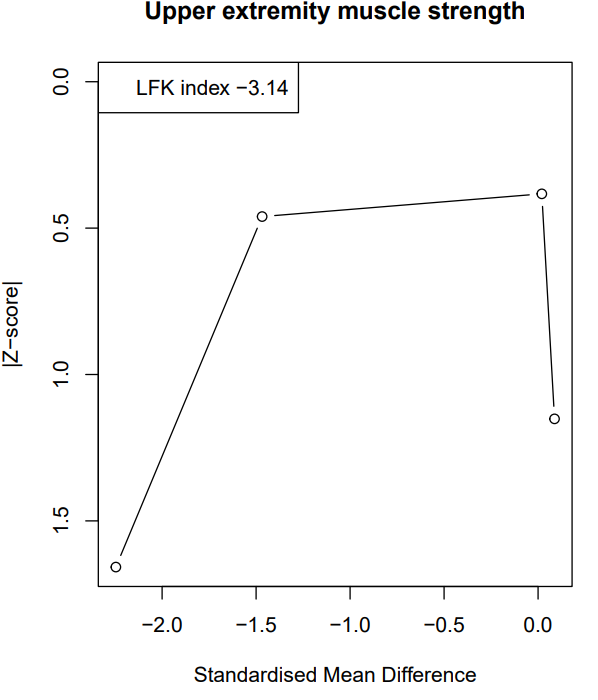

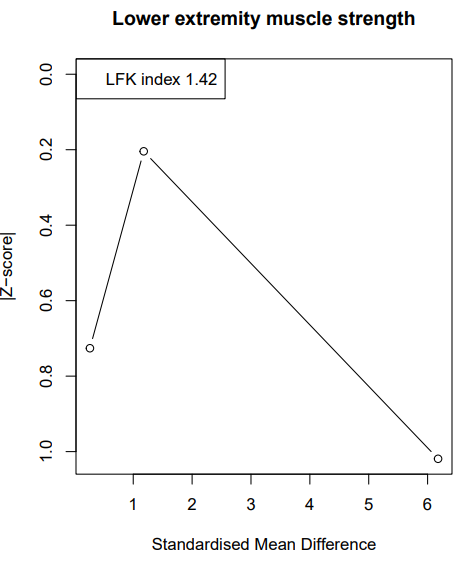

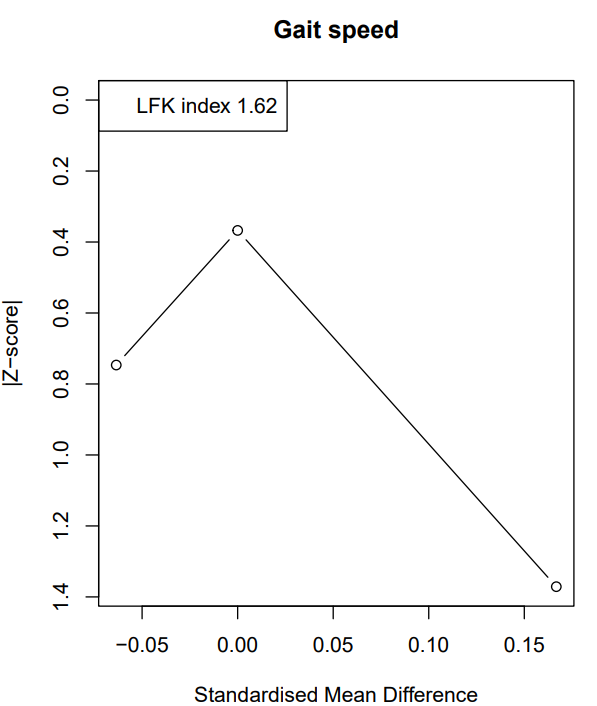


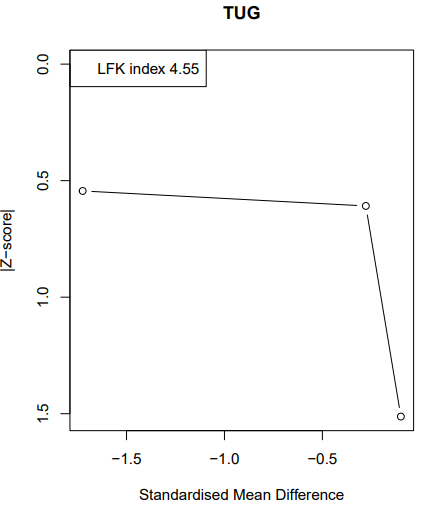

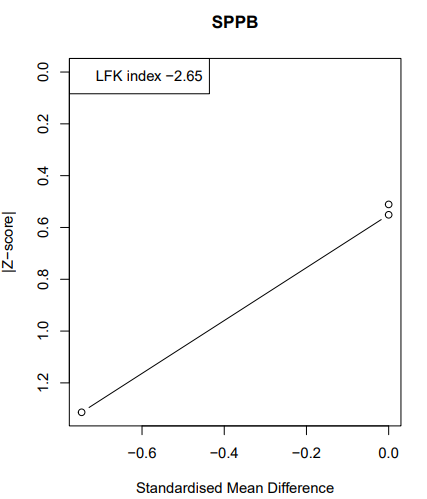

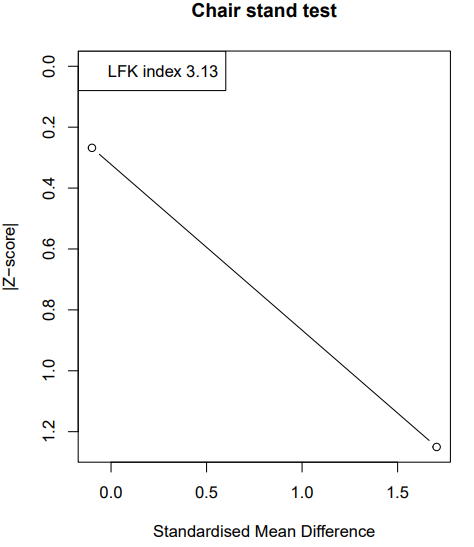


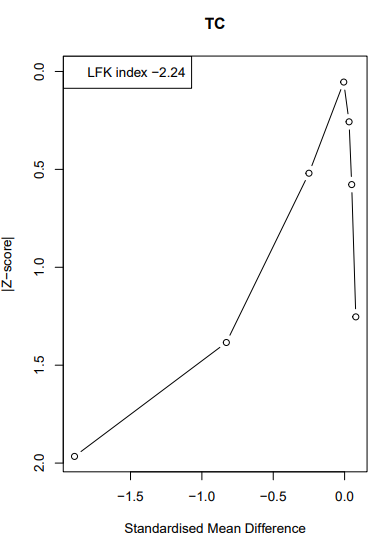

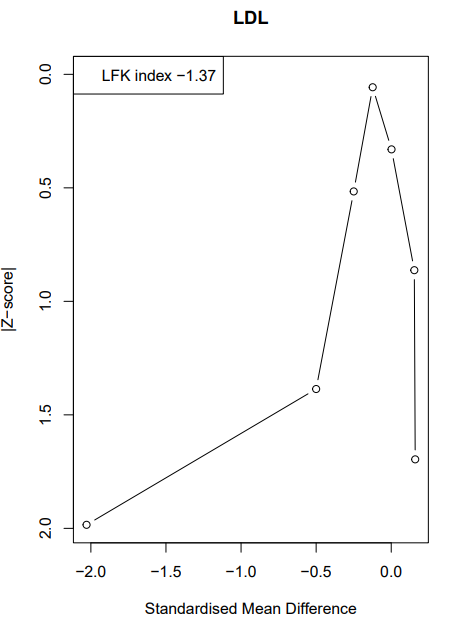

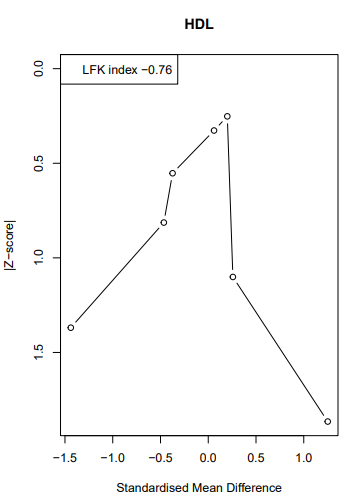

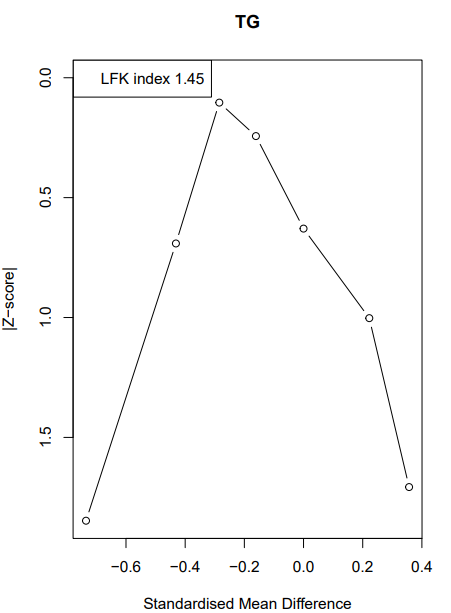


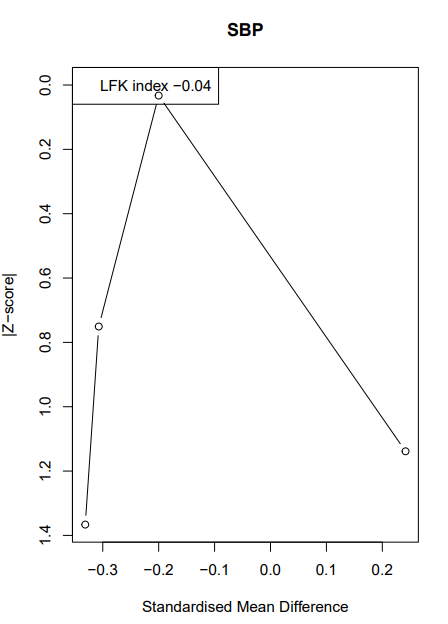

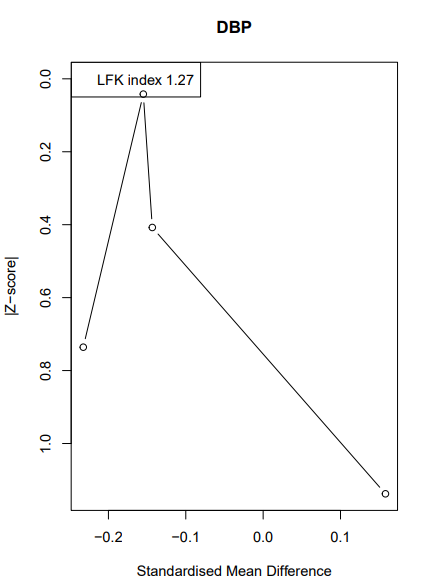

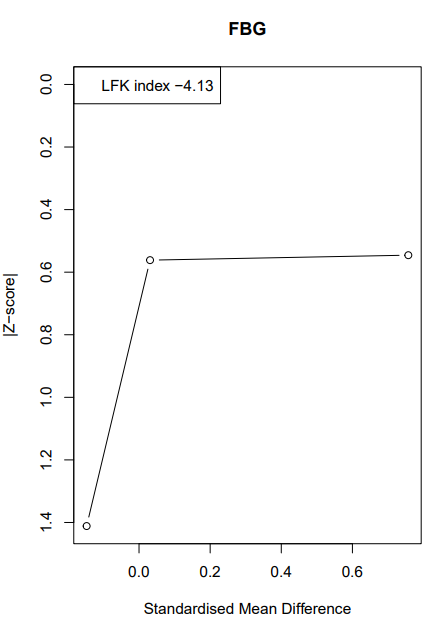


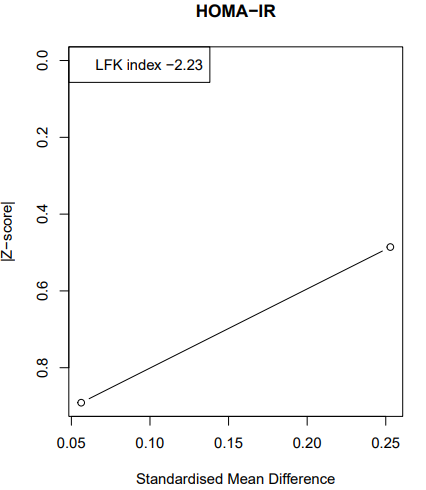

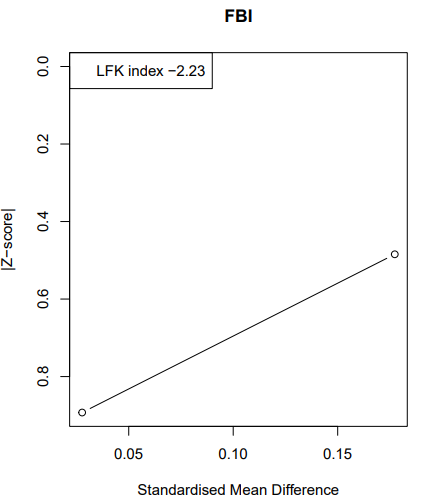


Supplemental Figure 2: Doi plots assessing small study effect due to publication bias for the studied outcomes.

Supplemental Table 8. GRADE evidence for long-term effect of Plant versus Animal Protein Supplementation on Body Composition, and Muscle Strength in Adults.

| Certainty assessment | | | | | | | № of patients | | Effect | | Certainty | Importance |
| --- | --- | --- | --- | --- | --- | --- | --- | --- | --- | --- | --- | --- |
| № of studies | Study design | Risk of bias | Inconsistency | Indirectness | Imprecision | Other considerations | Plant protein | Animal protein | Relative (95% CI) | Absolute (95% CI) |  |  |
| Lean body mass (follow-up: range 6 months to 31 months; assessed with: kg) | | | | | | | | | | | | |
| 9 | randomised trials | not serious | serious^a^ | not serious | serious^b^ | none | 200 | 165 | - | SMD 0.26 higher (0.68 lower to 1.21 higher) | ⨁⨁◯◯ Low^a,b^ |  |
| Fat mass (follow-up: range 6 months to 9 months; assessed with: Kg) | | | | | | | | | | | | |
| 8 | randomised trials | not serious | serious^a^ | not serious | serious^b^ | none | 169 | 134 | - | SMD 0.16 higher (0.58 lower to 0.90 higher) | ⨁⨁◯◯ Low^a,b^ |  |
| Total body mass (follow-up: range 6 months to 31 months; assessed with: kg) | | | | | | | | | | | | |
| 10 | randomised trials | not serious | serious^a^ | not serious | serious^b^ | none | 359 | 326 | - | SMD 0.37 higher (0.33 lower to 1.07 higher) | ⨁⨁◯◯ Low^a,b^ |  |

| Upper extremity muscle strength (follow-up: range 6 months to 24 months) | | | | | | | | | | | | |
| --- | --- | --- | --- | --- | --- | --- | --- | --- | --- | --- | --- | --- |
| 4 | randomised trials | not serious | serious^c^ | not serious | serious^d^ | publication bias strongly suspected^e^ | 188 | 124 | - | SMD 0.88 lower (1.99 lower to 0.22 higher) | ⨁◯◯◯ Very low^c,d,e^ |  |
| lower extremity muscle strength | | | | | | | | | | | | |
| 3 | randomised trials | not serious | serious^c^ | not serious | serious^d^ | publication bias strongly suspected^e^ | 139 | 82 | - | SMD 2.54 higher (1.07 lower to 6.14 higher) | ⨁◯◯◯ Very low^c,d,e^ |  |

CI: confidence interval; SMD: standardised mean difference

#### Explanations

a. Some heterogeneity exists despite explained by sensitivity and sub-group analysis

b. The confidence limit crosses the effect size of 0.5

c. little or no overlap of the confidence intervals of the effect estimates

d. Not enough samples (< 400) and the confidence limit crosses the effect size of 0.5

e. Due to small number of studies, publication bias may suspected

Supplemental Table 9. GRADE evidence for long-term effect of Plant versus Animal Protein Supplementation on parameters of physical performance in Adults.

| Certainty assessment | | | | | | | № of patients | | Effect | | Certainty | Importance |
| --- | --- | --- | --- | --- | --- | --- | --- | --- | --- | --- | --- | --- |
| № of studies | Study design | Risk of bias | Inconsistency | Indirectness | Imprecision | Other considerations | Plant protein | Animal protein | Relative (95% CI) | Absolute (95% CI) |  |  |
| Gait speed | | | | | | | | | | | | |
| 3 | randomised trials | not serious | serious^a^ | not serious | serious^b^ | publication bias strongly suspected^c^ | 73 | 64 | - | SMD 0.001 lower (0.34 lower to 0.34 higher) | ⨁◯◯◯  Very low^a,b,c^ |  |
| Timed Up and Go (follow-up: range 6 months to 24 months) | | | | | | | | | | | | |
| 3 | randomised trials | not serious | serious^a^ | not serious | serious^b^ | publication bias strongly suspected^c^ | 147 | 85 | - | SMD 0.74 lower (1.78 lower to 0.3 higher) | ⨁◯◯◯  Very low^a,b,c^ |  |
| Chair Stand test (follow-up: range 6 months to 6 months) | | | | | | | | | | | | |
| 2 | randomised trials | not serious | not serious | not serious | Serious^b^ | publication bias strongly suspected^c^ | 43 | 42 | - | SMD 0.75 higher (1.01 lower to 2.52 higher) | ⨁⨁◯◯ Low^b,c^ |  |
| SPPB (follow-up: range 6 months to 12 months) | | | | | | | | | | | | |
| 3 | randomised trials | not serious | serious^a^ | not serious | Serious^b^ | publication bias strongly suspected^c^ | 136 | 122 | - | SMD 0.22 lower (0.67 lower to 0.24 higher) | ⨁◯◯◯ Very low^a,b,c^ |  |

CI: confidence interval; SMD: standardised mean difference

#### Explanations

a. Some heterogeneity exists

b. Small sample size (< 400) and the confidence limit crosses the effect size of 0.5

c. Due to small number of studies, publication bias may be suspected

Supplemental Table 10. Long-term effect of Plant versus Animal Protein Supplementation on lipid profiles in Adults

| **Certainty assessment** | | | | | | | **№ of patients** | | **Effect** | | **Certainty** | **Importance** |
| --- | --- | --- | --- | --- | --- | --- | --- | --- | --- | --- | --- | --- |
| **№ of studies** | **Study design** | **Risk of bias** | **Inconsistency** | **Indirectness** | **Imprecision** | **Other considerations** | **Plant protein** | **Animal protein** | **Relative (95% CI)** | **Absolute (95% CI)** |  |  |
| **TC (follow-up: range 6 months to 31 months)** | | | | | | | | | | | | |
| 8 | randomised trials | not serious | serious^a^ | not serious | not serious | publication bias strongly suspected^b^ | 469 | 409 | - | SMD **0.38 lower** (0.88 lower to 0.13 higher) | ⨁⨁◯◯  Low ^a,b^ |  |
| **LDL (follow-up: range 6 months to 31 months)** | | | | | | | | | | | | |
| 8 | randomised trials | not serious | serious^a^ | not serious | not serious | publication bias strongly suspected^b^ | 469 | 409 | - | SMD **0.34 lower** (0.87 lower to 0.19 higher) | ⨁⨁◯◯  Low ^a,b^ |  |
| **HDL (follow-up: range 6 months to 31 months)** | | | | | | | | | | | | |
| 8 | randomised trials | not serious | serious^a^ | not serious | not serious | none | 469 | 409 | - | SMD **0.08 lower** (0.69 lower to 0.52 higher) | ⨁⨁⨁◯ Moderate^c^ |  |
| **TG (follow-up: range 6 months to 31 months)** | | | | | | | | | | | | |
| 8 | randomised trials | not serious | serious^a^ | not serious | not serious | publication bias strongly suspected^b^ | 469 | 409 | - | SMD **0.15 lower** (0.41 lower to 0.12 higher) | ⨁⨁◯◯ Low^a,b^ |  |

**CI:** confidence interval; **SMD:** standardised mean difference

#### Explanations

a. Considerable statistical heterogeneity

b. Doi plot showed small-study effect possibly consistent with publication bias

Supplemental Table 11. Long-term effect of Plant versus Animal Protein Supplementation on blood pressure, FBG, FBI, and HOMA-IR in Adults.

| Certainty assessment | | | | | | | № of patients | | Effect | | Certainty | Importance |
| --- | --- | --- | --- | --- | --- | --- | --- | --- | --- | --- | --- | --- |
| № of studies | Study design | Risk of bias | Inconsistency | Indirectness | Imprecision | Other considerations | Plant protein | Animal protein | Relative (95% CI) | Absolute (95% CI) |  |  |
| SBP (follow-up: range 6 months to 31 months) | | | | | | | | | | | | |
| 5 | randomised trials | not serious | serious^c^ | not serious | not serious | none | 343 | 347 | - | SMD 0.13 lower (0.40 lower to 0.13 higher) | ⨁⨁⨁◯ Moderate^c^ |  |
| DBP (follow-up: range 6 months to 31 months) | | | | | | | | | | | | |
| 5 | randomised trials | not serious | not serious | not serious | not serious | publication bias strongly suspected^b^ | 343 | 347 | - | SMD 0.10 lower (0.30 lower to 0.1 higher) | ⨁⨁⨁◯ Moderate^b^ |  |
| FBG (follow-up: range 6 months to 31 months) | | | | | | | | | | | | |
| 4 | randomised trials | not serious | serious^d^ | not serious | not serious | publication bias strongly suspected^b^ | 248 | 240 | - | SMD 0.24 higher (0.32 lower to 0.79 higher) | ⨁⨁◯◯ Low^a,b^ |  |
| FBI (follow-up: range 6 months to 6 months) | | | | | | | | | | | | |
| 2 | randomised trials | not serious | not serious | not serious | serious^a^ | publication bias strongly suspected^b^ | 95 | 97 | - | SMD 0.12 higher (0.16 lower to 0.4 higher) | ⨁⨁◯◯ Low^a,b^ |  |
| HOMA-IR (follow-up: range 6 months to 6 months) | | | | | | | | | | | | |
| 2 | randomised trials | not serious | not serious | not serious | serious^a^ | publication bias strongly suspected^b^ | 95 | 97 | - | SMD 0.18 higher (0.1 lower to 0.46 higher) | ⨁⨁◯◯ Low^a,b^ |  |

CI: confidence interval; SMD: standardised mean difference

#### Explanations

a. Small sample size (< 400) and the confidence limit crosses the effect size of 0.5

b. Due to small number of studies, publication bias may be suspected

c. substantial heterogeneity exists

d. Considerable statistical heterogeneity


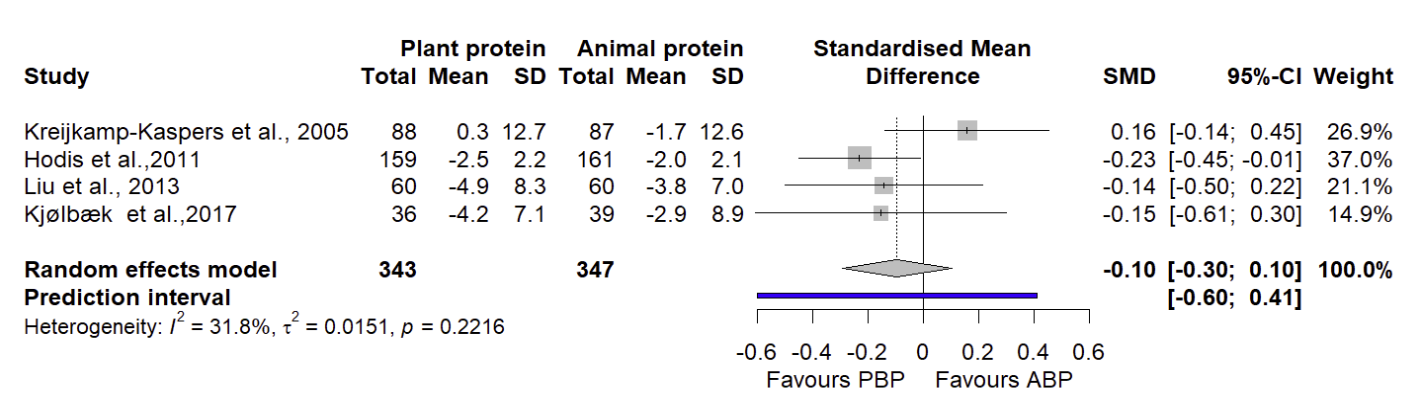


Supplemental Figure A. Forest plot of the long-term effect of PBP versus ABP supplementation on DBP in Adults, Using the Random Effects Model.


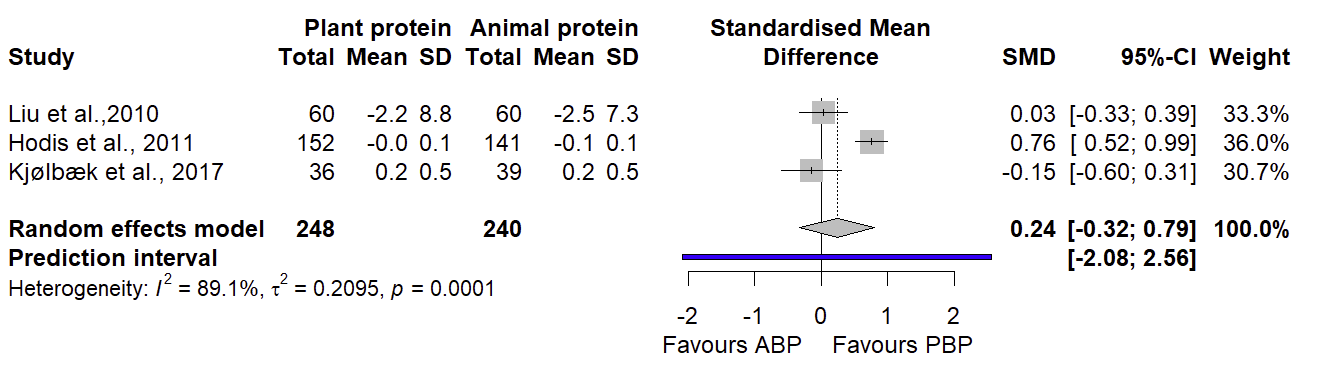


Supplemental Figure B. Forest plot of the long-term effect of PBP versus ABP supplementation on FBG in Adults, Using the Random Effects Model.


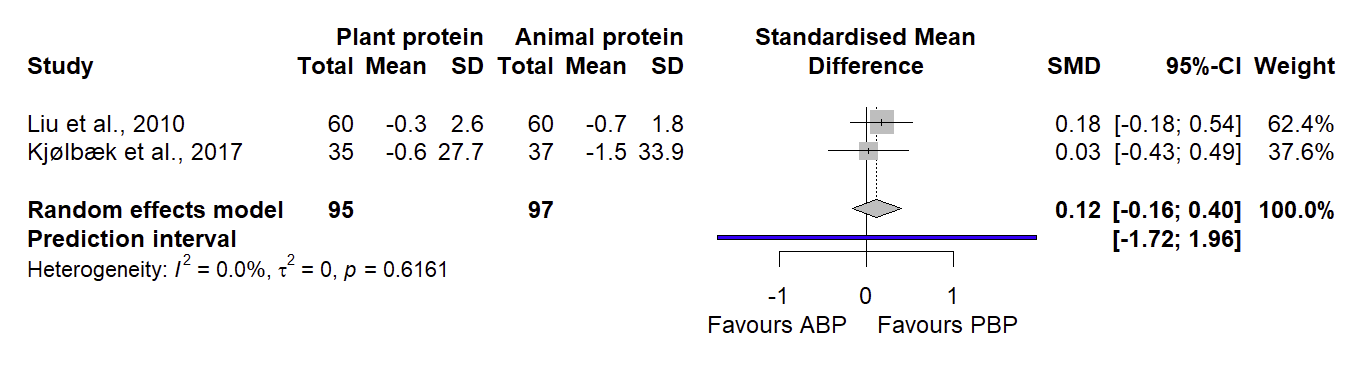


Supplemental Figure C. Forest plot of the long-term effect of PBP versus ABP supplementation on FBI in Adults, Using the Random Effects Model.


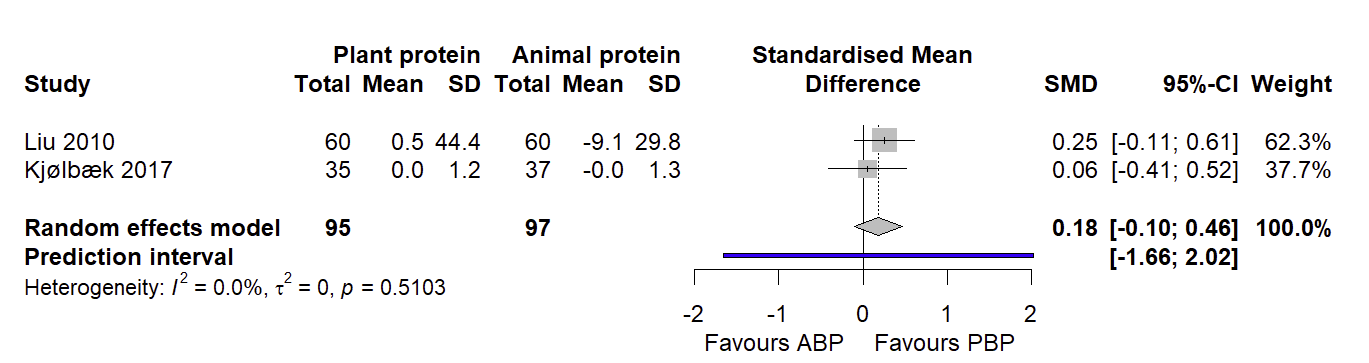


Supplemental Figure D. Forest plot of the long-term effect of PBP versus ABP supplementation on HOMA-IR in Adults, Using the Random Effects Model.
